# Targeting aberrant DNA methylation in mesenchymal stromal cells as a treatment for myeloma bone disease

**DOI:** 10.1038/s41467-020-20715-x

**Published:** 2021-01-18

**Authors:** Antonio Garcia-Gomez, Tianlu Li, Carlos de la Calle-Fabregat, Javier Rodríguez-Ubreva, Laura Ciudad, Francesc Català-Moll, Gerard Godoy-Tena, Montserrat Martín-Sánchez, Laura San-Segundo, Sandra Muntión, Xabier Morales, Carlos Ortiz-de-Solórzano, Julen Oyarzabal, Edurne San José-Enériz, Manel Esteller, Xabier Agirre, Felipe Prosper, Mercedes Garayoa, Esteban Ballestar

**Affiliations:** 1 Epigenetics and Immune Disease Group, Josep Carreras Leukaemia Research Institute (IJC), Badalona, 08916 Badalona, Barcelona Spain; 2 Chromatin and Disease Group, Cancer Epigenetics and Biology Programme (PEBC), Bellvitge Biomedical Research Institute (IDIBELL), 08908 L’Hospitalet de Llobregat, Barcelona Spain; 3 Centro de Investigación del Cáncer, IBMCC (Universidad de Salamanca-CSIC) and Hospital Universitario de Salamanca-IBSAL, 37007 Salamanca, Spain; 4 Imaging Platform, Center for Applied Medical Research (CIMA), University of Navarra, IDISNA, Ciberonc, 31008 Pamplona, Spain; 5 Small Molecule Discovery Platform, Molecular Therapeutics Program, Center for Applied Medical Research (CIMA), University of Navarra, 31008 Pamplona, Spain; 6 Division of Hemato-Oncology, Center for Applied Medical Research (CIMA), University of Navarra, IDISNA, Ciberonc, 31008 Pamplona Spain; 7 Josep Carreras Leukaemia Research Institute (IJC), Badalona, Barcelona, Catalonia Spain; 8 Centro de Investigacion Biomedica en Red Cancer (CIBERONC), Madrid, Spain; 9 Institucio Catalana de Recerca i Estudis Avançats (ICREA), Barcelona, Catalonia Spain; 10 Physiological Sciences Department, School of Medicine and Health Sciences, University of Barcelona (UB), Barcelona, Catalonia Spain

**Keywords:** Myeloma, Target identification, DNA methylation

## Abstract

Multiple myeloma (MM) progression and myeloma-associated bone disease (MBD) are highly dependent on bone marrow mesenchymal stromal cells (MSCs). MM-MSCs exhibit abnormal transcriptomes, suggesting the involvement of epigenetic mechanisms governing their tumor-promoting functions and prolonged osteoblast suppression. Here, we identify widespread DNA methylation alterations of bone marrow-isolated MSCs from distinct MM stages, particularly in Homeobox genes involved in osteogenic differentiation that associate with their aberrant expression. Moreover, these DNA methylation changes are recapitulated in vitro by exposing MSCs from healthy individuals to MM cells. Pharmacological targeting of DNMTs and G9a with dual inhibitor CM-272 reverts the expression of hypermethylated osteogenic regulators and promotes osteoblast differentiation of myeloma MSCs. Most importantly, CM-272 treatment prevents tumor-associated bone loss and reduces tumor burden in a murine myeloma model. Our results demonstrate that epigenetic aberrancies mediate the impairment of bone formation in MM, and its targeting by CM-272 is able to reverse MBD.

## Abstract (web summary)

Mesenchymal stromal cells (MSCs) have been shown to support multiple myeloma (MM) development. Here, MSCs isolated from the bone marrow of MM patients are shown to have altered DNA methylation patterns and a methyltransferase inhibitor reverts MM-associated bone loss and reduces tumour burden in MM murine models.

## Introduction

Multiple myeloma (MM) is an incurable hematological malignancy characterized by clonal expansion of plasma cells in the bone marrow (BM) that accounts for 1% of all cancers^1,2^. Nearly 90% of myeloma patients suffer from skeletal-related events during the course of the disease, including severe bone pain, hypercalcemia, pathological fractures, and spinal cord compression^3^, that not only affect the quality of life but also their overall survival^4^. Myeloma-associated bone disease (MBD) is characterized by an increase in bone-resorptive activity and number of osteoclasts (OCs), as well as impairment of bone-forming activity and differentiation of osteoblasts (OBs), which ultimately lead to the development of osteolytic lesions^5^.

In most cases, symptomatic myeloma is preceded by sequential asymptomatic stages of monoclonal gammopathy of undetermined significance (MGUS) and smoldering myeloma (SMM), with increasing BM plasmocytosis and monoclonal component as well as augmented risk of progression to active MM^6,7^. The biological behavior and clinical outcome of MM are partly dependent on genetic and epigenetic abnormalities of tumor subclones that arise from MGUS and SMM stages^8^. However, the clinical stability of MGUS cases, despite displaying shared genetic lesions with MM cells, suggests that the BM microenvironment may critically modulate disease progression^6,9,10^. In this regard, it has been widely shown that a complex and bidirectional relationship exists between MM cells and the BM niche, which results in oncogenesis support, anemia, immunosuppression, and uncoupling of the bone remodeling process^11^.

Mesenchymal stromal cells (MSCs) are an essential cell type in the formation and function of the BM microenvironment, being the progenitors of bone-forming OBs, adipocytes, and chondroblasts, as well as the hematopoietic-supporting stroma components of the BM^12^. It is well-documented that BM-derived MSCs from MM patients contribute to MM progression (reviewed in ref. ^11^) and show an impaired ability to differentiate into OBs^13,14^. Moreover, MM-MSCs are considered inherently abnormal, as their dysfunctionality remains even following ex vivo culture in the absence of MM cells^15^. Furthermore, bone lesions persist in many MM patients even after therapeutic remission, suggesting a long-term defect in MSCs that inhibit their ability to properly differentiate into functional OBs^16^.

Previous studies described that MSCs from MM patients are cytogenetically normal^17,18^, but show alterations in their transcriptional^13,19^ and proteomic^11^ profiles even in the absence of myeloma cell interaction. This suggests that epigenetic mechanisms could be governing the tumor-promoting functions of MSCs and their prolonged OB suppression in MM. In fact, Adamik and colleagues^20^ reported abnormal recruitment of chromatin remodelers in MSCs from myeloma patients, contributing to the transcriptional repression of Runx2, a master regulator of OB differentiation. Yet, there is a lack of information about DNA methylation-related mechanisms that may contribute to MM progression and subsequent bone defects. DNA methylation is an essential epigenetic modification involving the addition of a methyl group to the 5-carbon of the cytosine ring by a family of DNA methyltransferase (DNMT) enzymes^21^, which has been described to play a critical role in MSC lineage determination^22^, as well as in tumor progression and immunosuppression in other cancer types^23^.

In this study, we identify DNA methylation alterations in MSCs of MM patients mediated by MM cells resulting in the dysregulation of osteogenesis, and this is reversed by the treatment with CM-272, a dual inhibitor of DNMTs and the histone methyltransferase G9a.

## Results

### BM-derived MSCs of distinct MM stages exhibit altered DNA methylation profiles

**Fig. 1:**
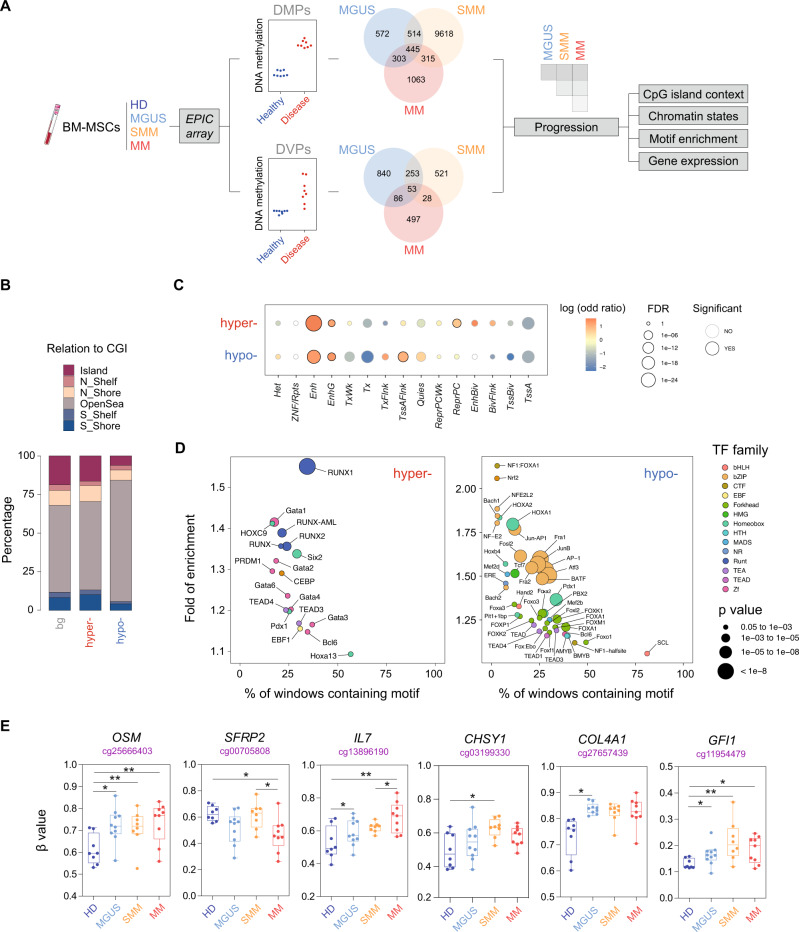
High-throughput stepwise DNA methylation changes in BM-derived MSCs associated with MM progression. **A** Workflow depicting the methodological approach for selecting DNA methylation changes in bone marrow-derived mesenchymal stromal cells (BM-MSCs) from monoclonal gammopathy of undetermined significance (MGUS; *n* = 10), smoldering myeloma (SMM; *n* = 8), and multiple myeloma (MM; *n* = 9) patients versus healthy controls (HD; *n* = 8). An example of a CpG site experiencing increased mean (differentially methylated position, DMP) or variance (differentially variable position, DVP) in the disease versus the control condition is shown. Venn diagrams show the number of DMPs or DVPs resulting from each comparison. **B** Distribution of DNA methylation changes in relation to CpG islands (CGI), including shores (south, S; north, N), shelves (south, S; north, N), and open sea regions for differentially hyper- or hypomethylated CpG sites. **C** Enrichment analysis of differentially hyper- and hypomethylated CpG sites located in different genomic regions, annotated by 15 chromHMM states. Color scale refers to log odd ratio and circle size refers to *p*-value significance. **D** Bubble plot representation of HOMER transcription factor (TF) motif enrichment analysis of differentially hyper- and hypomethylated CpGs in MSCs during MM progression (left and right panel, respectively). Color range depicts different transcription factor families and circle size refers to *p*-value significance. **E** Box plots showing *β*-values from DMPs obtained from the EPIC array in MSCs from healthy donors and MGUS, SMM, and MM patients of relevant genes involved in the pathogenesis of MM and associated bone disease. HD is represented in dark blue, MGUS in light blue, SMM in orange, and MM in red. eBayes-moderated ANOVA *t*-test was performed to calculate statistical significance (*p < 0.05, **p < 0.01, and ***p < 0.005).

We first obtained genome-wide DNA methylation profiles of BM-derived MSCs isolated at different stages of MM (newly diagnosed MGUS, high-risk SMM, and MM) and healthy controls. DNA methylation changes were identified using two different statistical approaches (Fig. 1A): (i) detection of differentially methylated CpG positions (DMPs) based on differences in DNA methylation means between the patient (MGUS, SMM, and MM) and healthy MSCs (Δ*β* ≥ 0.1 and **p < 0.01) (Supplementary Data 1); and (ii) detection of differentially variable CpG positions (DVPs) based on differences in variance of DNA methylation levels (*q*val < 0.05 and *p < 0.05) between the sample groups (Supplementary Data 2). In regards to DMPs, the largest number of altered CpGs was found in MSCs from patients of the SMM stage compared to healthy donors (Supplementary Fig. 1A, B). On the other hand, we observed the highest number of DVPs in comparison to healthy donors in MSCs isolated from MGUS followed by SMM and MM patients (Supplementary Fig. 1C, D), supporting the notion that these stochastic and heterogeneous DNA methylation patterns are associated with early stages of carcinogenesis, as previously reported^24,25^. We also observed that the majority of identified DMPs and DVPs are disease stage-specific, although the asymptomatic stages showed a moderate proportion of overlap (Fig. 1A).

Given that myeloma is a multi-stage disease, we then analyzed the accumulative changes of DNA methylation associated with MM progression by selecting DMPs (Supplementary Data 3) and DVPs (Supplementary Data 4) that were found either only in the MM stage, shared by SMM and MM and in all three stages (Fig. 1A). With this analysis, we identified 872 hyper- and 951 hypomethylated DMPs, and 260 hyper- and 318 hypomethylated DVPs.

Analyzing the distribution of MM progression-associated CpGs in relation to CpG islands (CGI), we observed a significant enrichment of CpGs in open sea regions in the hypomethylated DMP data set (Fig. 1B) and in CpG islands in the hypermethylated DVP data set (Supplementary Fig. 1E). Utilizing publicly available chromatin state maps of BM-derived MSCs from healthy individuals^26^, we found a significant enrichment of both hyper- and hypomethylated DMPs sites that correspond to enhancers (Fig. 1C). In addition, we observed an enrichment in flanking transcription start sites (TSS) in the hypomethylated set, and regions repressed by Polycomb Group (PcG) in hypermethylated CpGs (Fig. 1C). On the other hand, hypermethylated DVPs were enriched in TSSs, bivalent regions, and regions repressed by Polycomb (Supplementary Fig. 1F).

To determine whether these MM progression-associated loci shared any common DNA elements, we performed a search for enriched transcription factor (TF)-binding sites in these regions using the HOMER algorithm^27^. We observed a significant over-representation of binding sites for the Runt and Tead family in differentially hypermethylated DMPs associated with MM progression (**p < 0.01; Fig. 1D). These results suggest that key transcription factors involved in the upregulation of osteogenic genes, such as *RUNX2*^28^ or *TEAD2*^29^, may participate in aberrant DNA hypermethylation. Since DNA methylation has been originally linked to transcriptional repression, these results suggested that the hypermethylation of these regions could compromise the ability of MSCs to undergo proper OB differentiation. On the other hand, DMP sites that experienced aberrant DNA hypomethylation were highly enriched in binding motifs of the bZip and Homeobox families (**p < 0.01; Fig. 1D). In this respect, the loss of DNA methylation could be selectively driving the occupancy of TF that have been reported as negative regulators of OB differentiation such as HOXA2^30^ and ATF3^31^. In addition, we observed transcriptional deregulation of some members of these TF families using expression array data from BM-derived MSCs of healthy controls, MGUS, SMM, and MM patients. Some of these TFs were specifically downregulated in MSCs of active myeloma (*RUNX2* and *TEAD2*), others were already downregulated in precursor myeloma stages (*HOXC9* and *CEBPD*), whereas other TFs, including *HOXA2* and *ATF3*, did not change their expression in any myeloma stages (Supplementary Fig. 1G). In all, these findings suggested that MM progression-associated DNA methylation changes in MSCs might be mediated by the sequential activity of specific TF families, which are also functionally deregulated in MM^32^. Furthermore, other genes that play important roles in the pathophysiology of MM (such as the cytokines *IL6* and *OSM*) and associated MBD (secreted factors such as *RANKL*, *SFRP2*, *IL7*, *CHSY1*, *COL4A1*, and the transcriptional repressor *GFI1*) were also found to alter their DNA methylation levels (Fig. 1E and Supplementary Fig. 1H).

### Aberrant DNA methylation is associated with differential Homeobox gene expression in MSCs at different MM stages

**Fig. 2:**
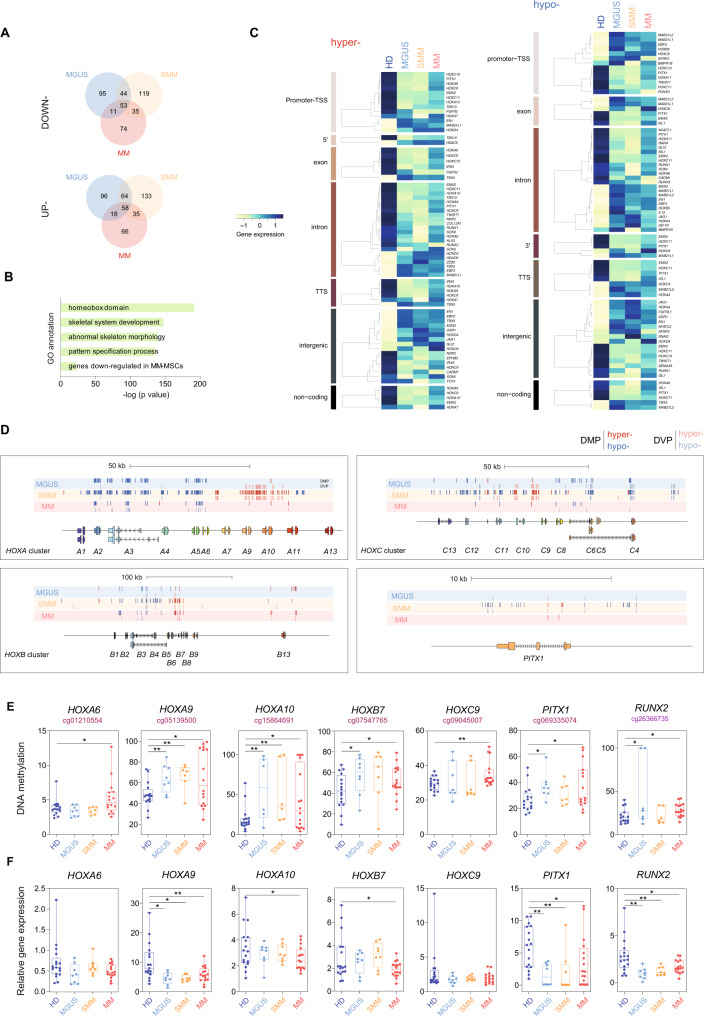
DNA methylation changes are associated with differential gene expression of Homeobox genes in MSCs from MGUS, SMM and MM patients. **A** Venn diagrams showing differentially methylated or downregulated (upper) and upregulated (lower panel) genes when comparing MGUS (blue), SMM (orange), and MM (red) samples with healthy individuals. **B** Gene ontology (GO) enrichment analysis of CpG sites undergoing DNA methylation and gene expression changes in MSCs of patients compared to controls using the GREAT online tool. A binomial test was performed to calculate statistical significance. **C** Heatmaps showing gene expression of Homeobox and other OB-related genes associated with differentially hyper- (left) or hypomethylated (right) CpG sites. Heatmaps are grouped according to the genomic location (promoter, TSS, 5′, exon, intron, intergenic, 3′ or non-coding region) of analyzed CpG sites. Color scale ranging from light yellow to dark blue represents low to high expression levels. **D** Scheme depicting differentially methylated and variable CpG sites located in the Homeobox genes (*HOX-A*, *-B,* and -*C clusters*) and *PITX1*. Dark blue lines indicate hypomethylated DMPs, light blue lines indicate hypomethylated DVPs, dark red lines indicate hypermethylated DMPs and light red lines indicate hypermethylated DVPs associated with MGUS, SMM, and MM condition. **E** DNA methylation analysis by pyrosequencing of selected CpGs located at the promoter regions and **F** gene expression of *HOXA6, -A9, -A10, -B7, -C9, PITX1,* and *RUNX2* in MSCs from healthy controls (dark blue; *n* = 17), MGUS (light blue; *n* = 8), SMM (orange; *n* = 8), and MM (*n* = 16) patients. Gene expression was normalized against *RPL38*. Box plots represent median ± IQR and whiskers represent maximum and minimum. Statistical significance was calculated using unpaired two-tailed Student *t*-tests (*p-value < 0.05, **p-value < 0.01, and ***p-value < 0.005).

To further investigate the relationship between differential DNA methylation and gene expression, we mapped the DMPs to the most proximal gene. Using expression array data from BM-derived MSCs of healthy controls, MGUS, SMM, and MM patients (Supplementary Data 5), differential expression of DMP-associated genes was identified using a cutoff of *p < 0.05 comparing MGUS/SMM/MM to healthy controls for both DNA methylation and gene expression (Fig. 2A and Supplementary Data 6). Gene ontology (GO) analysis revealed that the genes displaying both differential methylation and expression were enriched in functional categories important in cell fate commitment and bone phenotype (Fig. 2B). The most enriched functional category corresponded to genes from the Homeobox family. Within the Homeobox family, we found the subset of *Hox* genes that encodes a large family of highly conserved TFs responsible for driving the correct differentiation of MSCs^33^, namely genes belonging to the HOXA-to-D clusters. Furthermore, we observed a significant enrichment in genes reported to be downregulated in MM-MSCs (Fig. 2B)^13^. Integration of methylation and gene expression data corresponding to the Homeobox and bone formation-related genes revealed that both DNA hyper- and hypomethylation events were associated with both downregulation and upregulation of gene expression in different genomic locations (Fig. 2C). Specifically, hypermethylated genes that showed a reduced expression in patient MSCs include positive regulators of OB differentiation such as *RUNX2* or *NRP2*^34^ (Fig. 2C and Supplementary Table 1) In contrast, negative regulators of osteogenesis such as *SFRP2*^35^ or *NFATC2*^36^ were hypomethylated and consequently upregulated in patient MSCs (Fig. 2C). In all, these factors could potentially contribute to impaired osteoblastogenesis associated with bone disease in MM and this is summarized in Supplementary Table 1.

Upon a closer inspection of several Homeobox-associated genomic regions, we observed a negative correlation between DNA methylation of promoters and gene expression. Specifically, the HOXA gene cluster showed aberrant hypomethylation at the *HOXA4* promoter, and its gene expression was upregulated at different disease stages. Conversely, gene promoters of *HOX-A6, -A7, -A9, -A10,* and *-A11* displayed hypermethylation and these genes were downregulated in MGUS/SMM/MM (Fig. 2D and Supplementary Fig. 2A). A similar pattern of an inverse association between methylation and expression was observed in the HOXB and HOXC gene cluster, where *HOXB5, -C5,* and *-C8* were aberrantly hypomethylated and upregulated, whereas *HOXC9, -C10,* and *-C11* were hypermethylated and downregulated in patients (Fig. 2D and Supplementary Fig. 2A). Other Homeobox genes such as *TBX5*, *PITX1*, or *EMX2* were also reported as regulators of bone formation^37–39^ and showed an association between DNA methylation at gene promoter and gene expression (Fig. 2D and Supplementary Fig. 2A, B).

We then validated the aforementioned DNA methylation and gene expression changes in an independent cohort of BM-derived MSCs from different MM disease stages by pyrosequencing and real-time quantitative PCR. Among the differentially methylated genes of the Homeobox family, we selected *HOXA2*, *-A4, -A6, -A9, -A10, -B7, -C9*, -*C10,* and *PITX1* on the basis of their reported role in MSC pluripotency (Fig. 2E, F and Supplementary Fig. 2C, D)^40^. Furthermore, we validated differentially methylated genes with osteogenic roles in the myeloma context, including *RUNX2* and *IBSP* (Fig. 2E, F and Supplementary Fig. 2C, D). In most cases, we observed that DNA methylation negatively correlated with gene expression.

### Healthy MSCs change their DNA methylation profile to one partially resembling that of MSCs from MM patients upon interaction with MM plasma cells

**Fig. 3:**
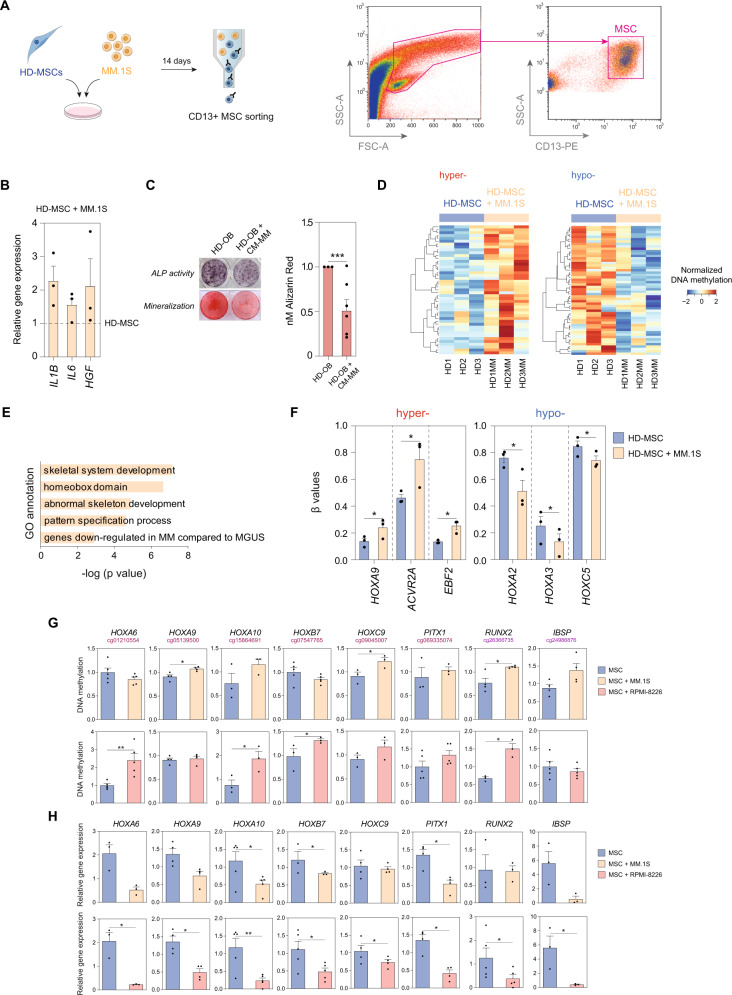
MSCs from healthy donors recapitulate DNA methylation changes observed in MSCs from MM patients upon interacting with MM plasma cells. **A** Scheme depicting workflow (left panel) and sorting strategy (right panel) for selecting CD13 + MSCs after 14 days of co-culture with the MM.1S cell line. **B** Gene expression analysis of *IL1B*, *IL6,* and *HGF*, normalized against *RPL38*, of the hMSC-TERT cell line (HD-MSC) co-cultured with the MM.1S cell line for 14 days. Expression levels were normalized against MSCs in monoculture. Data are represented as the mean ± SEM from three independent experiments. **C** ALP activity and matrix mineralization were assessed in differentiated osteoblasts from HD-MSCs in the presence (HD-OB + CM-MM) or absence (HD-OB) of conditioned media from the MM.1S cell line (CM-MM). Representative images of each experimental condition are shown. Barplot represents the mean ± SEM from six independent experiments, and a paired two-tailed *t*-test was performed to evaluate statistical significance (***p < 0.005). **D** Heatmap showing differentially methylated CpG sites (eBayes-moderated paired *t*-test *p < 0.05) in sorted HD-MSCs (three independent donors) in monoculture (HD1-3) or co-cultured with the MM.1S cell line (HD1-3MM) for 14 days that overlaps with previously identified DMPs. The color scale from blue to red represents low to high methylation levels. **E** GO enrichment analysis of DMPs in HD-MSCs co-cultured with the MM.1S cell line overlapping with myeloma-associated DMPs analyzed using the GREAT online tool. *p*-values were calculated using a binomial test. **F** Bar plots showing *β*-values obtained from the DNA methylation array presented in **D**, representing mean and ±SEM of three independent experiments. **G** DNA methylation and **H** gene expression levels of DMPs validated in HD-MSCs monoculture (blue) or co-cultured with MM.1S (orange) or RPMI-8226 (red) cell lines as indicated. Gene expression data were normalized against *RPL38* and all data were normalized against HD-MSC monoculture. Statistical significance was calculated using paired one-tailed Student *t*-tests and bar plots represent mean ± SEM of 3–5 independent experiments (*p-value < 0.05, **p-value < 0.01, and ***p-value < 0.005).

To address the potential contribution of MM cells in mediating aberrant DNA methylation changes in MSCs, we evaluated whether the epigenetic changes observed in MM-MSCs could be mimicked in vitro by direct contact of healthy MSCs with MM cells. Thus, we co-cultured BM-derived MSCs from healthy donors with the human MM cell line MM.1S for 2 weeks Subsequently, MSCs were sorted by CD13^+^ expression and subjected to DNA methylation analysis (Fig. 3A).

Under these conditions, MM.1S cells were able to induce the expression of genes known to be upregulated in MM-MSCs (*IL1B, IL6,* and *HGF*) in HD-MSCs compared with mono-cultured HD-MSCs (Fig. 3B). Additionally, we validated the inhibitory effect of MM cells in MSC-to-OB differentiation and observed a decrease in both ALP activity and OB mineralization in OBs differentiated in the presence of conditioned media from the MM.1S cell line as compared to OBs differentiated alone (Fig. 3C).

We then investigated the DNA methylation profiling of MSCs from healthy donors generated upon interaction with MM cells. We observed that 142 CpGs that change their methylation levels upon co-culture with MM.1S cells were shared with aberrant DNA profiles found in MSCs isolated from MGUS/SMM/MM patients (Fig. 3D and Supplementary Data 7). Although this accounted for a small percentage of DMPs identified in MM patient MSCs, GO analyses revealed enrichment in Homeobox genes and categories related to bone formation, similar to that observed in primary patient MSCs (Fig. 3E). Specifically, we found that healthy MSCs exposed to MM cells underwent gains (*HOXA9, ACVR2A, EBF2*) and losses (*HOXA2, HOXA3, HOXC5*) of DNA methylation in the direction of those observed for MSCs from myeloma patients (Fig. 3F).

Moreover, we validated the DNA methylation and gene expression changes in healthy MSCs driven by co-culture with MM.1S with another MM cell line, RPMI-8226. Here, we observed a similar effect of co-culture with RPMI-8226, in which there was a clear inhibition of Homeobox and osteogenic gene expression coupled with hypermethylation of these loci (Fig. 3G, H).

Altogether, these results support the notion that MM cells not only are capable of inducing changes in the global methylome of MSCs but also have a significant impact at specific osteogenic loci.

### Dual targeting of DNMTs and G9a restores Homeobox gene expression in vitro and promotes osteogenic differentiation of mesenchymal precursors

**Fig. 4:**
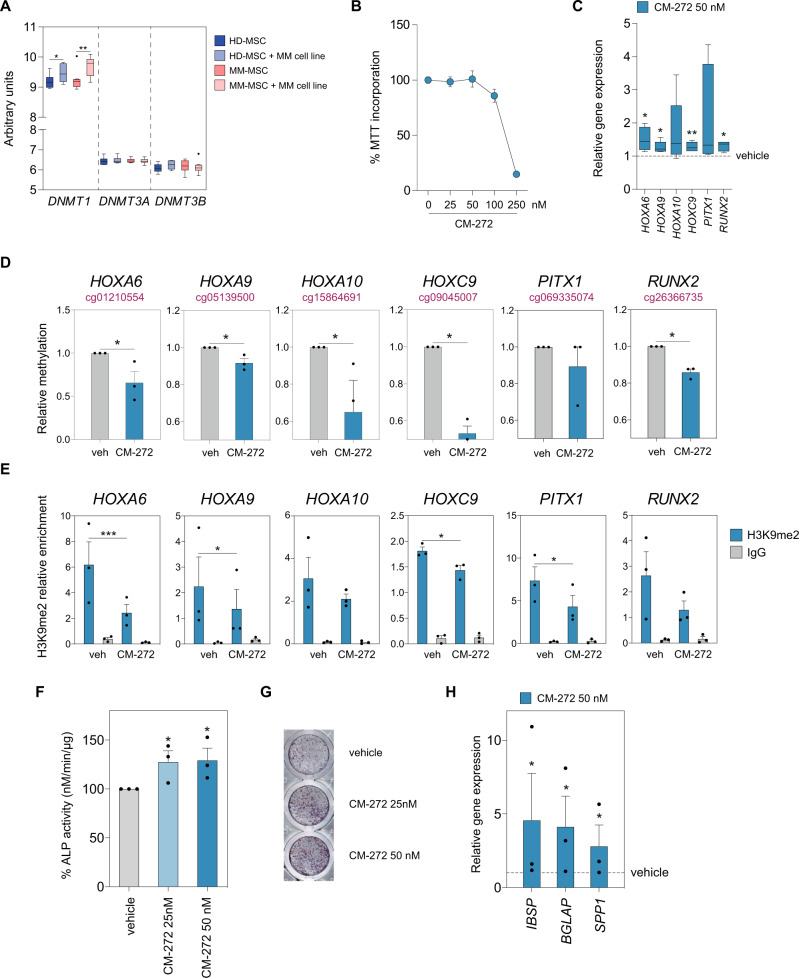
CM-272 treatment reactivates Homeobox gene expression and promotes the osteogenic differentiation of MSCs from MM patients. **A** Expression of DNA methyltransferases 1, 3A, and B (*DNMT1, -3A*, and *-3B*) in HD and MM-MSCs co-cultured for 24 h with the MM.1S cell line comparing with monoculture as assessed by the GeneChip Human Gene 1.0 ST Array. Box plots present mean ± SEM for 8 healthy donors and 9 MM patients. *p-*values were calculated by paired two-tailed Student *t*-test (*p < 0.05, **p < 0.01). **B** MSCs from MM patients were treated with the indicated doses of CM-272 for 72 h and subjected to MTT assay for viability. Mean and SEM are indicated on the line chart from 3 independent experiments. **C** Real-time RT-PCR was performed to determine the expression of hypermethylated Homeobox genes (*HOX-A6, -A9, -A10, -C9, PITX1, RUNX2*) in MM-MSCs treated with vehicle or 50 nM of CM-272 for 7 days. Box plots represent median ±IQR, with whiskers representing minimum and maximum, of 9 independent experiments. A paired two-tailed Student’s *t*-test was performed to calculate statistical significance (*p < 0.05, **p < 0.01). **D** DNA methylation analysis by pyrosequencing of selected CpGs located at the promoter regions of Homeobox genes in MM-MSCs treated with vehicle (gray) or CM-272 (blue) for 7 days. Bar plots present mean ± SEM for 3 independent experiments and paired two-tailed Student’s *t*-tests were performed (*p < 0.05). **E** ChIP assays showing the H3K9me2 (blue) enrichment at the promoter regions of Homeobox genes in MM-MSCs treated with vehicle or CM-272 for 7 days. IgG (gray) was used as a negative control. Data are shown as a relative enrichment of the bound fraction with respect to the input DNA. Bar plots present mean ± SEM for 3 independent experiments and paired two-tailed Student’s *t*-tests were performed (*p < 0.05, **p < 0.01, and ***p < 0.005). ALP activity was assessed in MM-MSCs (*n* = 3) cultured in osteogenic media in the presence of 25 nM (light blue) and 50 nM (dark blue) of CM-272, compared to vehicle (gray), by **F** p-NPP hydrolysis and **G** NBT-BCIP. **H** Expression of osteoblastogenesis markers *IBSP* (bone sialoprotein), *BGLAP* (osteocalcin) and *SPP1* (osteopontin) was checked by qRT-PCR in MM-MSCs cultured in osteogenic media in the absence (vehicle) or presence of CM-272. For **F** and **H** data are shown as mean values ± SEM from three different experiments. Statistically significant tests (paired two-tailed Student’s *t*-tests) are represented as *p < 0.05, **p < 0.01, and ***p < 0.005 between vehicle and CM-272 condition.

Gene expression analysis of DNMTs in MSCs from HD and MM patients co-cultured with MM cells obtained from a previous study^41^ showed an aberrant upregulation of the DNA methyltransferase DNMT1 (Fig. 4A). DNMT1 interacts with the methyltransferase G9a to coordinate DNA and H3K9 methylation during cell replication^42^ promoting transcriptional silencing of target genes. Moreover, G9a can suppress transcription by inducing DNA methylation in addition to its activity as a chromatin remodeler^43^. In this regard, we hypothesized that the dual inhibition of DNMT1 and G9a could reactivate hypermethylated and silenced genes of MSCs from MM patients preserving their osteogenic potential and therefore preventing myeloma-associated bone loss. Thus, we utilized a dual inhibitor of DNMTs and G9a, termed CM-272, which has been previously described to have a potent therapeutic response, both in vitro and in vivo, in other neoplasias^44–47^.

We first checked the effect of CM-272 on the cell viability of mesenchymal progenitors and we selected a dose (50 nM) with no significant toxicity in order to perform further experiments (Fig. 4B). CM-272 treatment was able to restore the expression of Homeobox genes (*HOX-A6, -A9, -A10, -C9, PITX1,* and *RUNX2*) that were epigenetically repressed in MSCs from MM patients (Fig. 4C). Mechanistically, we observed a loss of DNA methylation in the promoter region of the majority of the aforementioned genes after CM-272 treatment in MM-MSCs (Fig. 4D). We then checked the levels of the inactive chromatin mark H3K9me2, a hallmark of methyltransferase G9a activity, at these gene promoters upon CM-272 treatment. The chIP-qPCR analysis showed a decrease in H3K9me2 levels at the promoter regions of Homeobox genes after CM-272 treatment (Fig. 4E). Taken together, our results suggest that CM-272 acts in vitro by inhibition of both DNMT and G9a methyltransferase activity.

Next, we addressed whether targeting DNMT and G9a may have a role in regulating osteogenic differentiation. For this purpose, we cultured MSCs from myeloma patients in osteogenic media to obtain differentiated OBs in the presence or absence of CM-272. As observed in Fig. 4F, G, CM-272 was able to increase ALP activity in early-stage OBs. Furthermore, CM-272 treatment was able to upregulate the relative expression of several late bone formation markers (namely, bone siaploprotein, osteopontin, and osteocalcin) in MSCs from myeloma patients (Fig. 4H).

**Fig. 5:**
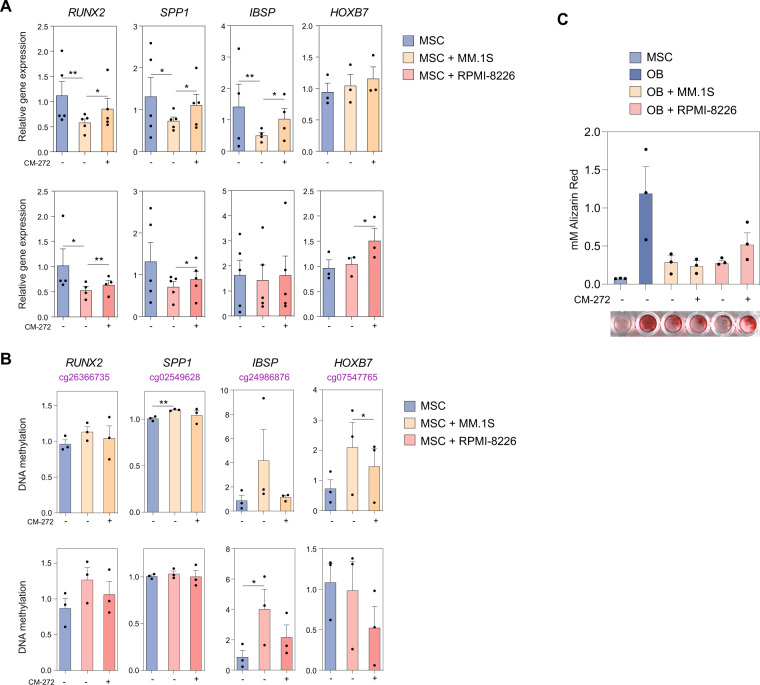
CM-272 restores the gene expression levels and suppression of mineralization in MSCs from healthy donors exposed to MM cells. HD-MSCs (MSC; blue) were co-cultured with MM.1S (orange) or RPMI-8226 (red) cell lines separated by a transwell system in the presence (darker shade) or absence (lighter shade) of CM-272. **A** Relative expression and **B** DNA methylation of genes *RUNX2*, *SPP1*, *IBSP,* and *HOXB7* was assessed. Gene expression was by normalization against *RPL38*. Bar plots represent mean ± SEM of 3–5 independent experiments and statistical significance was calculated by paired one-tailed Student’s *t*-test (*p < 0.05, **p < 0.01). **C** Mineralization was assessed by alizarin red staining in differentiated OBs from HD-MSCs co-cultured with MM.1S or RPMI-8226 cell lines in the presence or absence of CM-272. Representative micrographs show matrix mineralization by alizarin red staining of corresponding differentiated OBs. Data are represented as the mean ± SEM from three independent experiments and paired two-tailed Student’s *t*-tests were performed (*p < 0.05, **p < 0.01).

Previous research has described that MM cells exert their effect on MSCs through both direct cell–cell contact and soluble factor mechanisms^11,48^. Our main data show that direct myeloma-MSC co-culture conditions are able to induce changes in the MSC methylome; however, it would be of particular interest to investigate whether MM cells may also mediate the same changes only through soluble factor mechanisms. For this purpose, utilizing a transwell system avoiding contact between both cell types, we observed that soluble factor secreted by MM.1S and RPMI-8226 cell lines were sufficient to change the expression of several OB-relevant genes in healthy MSCs, including *RUNX2*, *SPP1*, *IBSP*, and *HOXB7* (Fig. 5A), concordantly to direct co-culture. Furthermore, treatment with the dual inhibitor CM-272 was able to partially reverse those changes in gene expression mediated by soluble factors secreted by MM cells (Fig. 5A). Changes in gene expression were accompanied by inverse changes in DNA methylation in some of the genes (*IBSP, HOXB7*) (Fig. 5B), which were also observed to at least be partially reversed by CM-272. However, for some other genes such as *RUNX2* and *SPP1*, transwell co-culture with MM cells induced minimal effect on DNA methylation, suggesting that direct cell–cell contact may be required. Moreover, CM-272 was able to partially reverse the MM cell lines-mediated inhibitory effect on OB mineralization (Fig. 5C). Altogether, these results suggest that MM cells at least partially exert its effects on MSCs through secretory mechanisms, and treatment with CM-272 was able to reserve these effects through the inhibition of DNA methylation.

### CM-272 not only controls tumor burden but also prevents the myeloma-associated bone loss

**Fig. 6:**
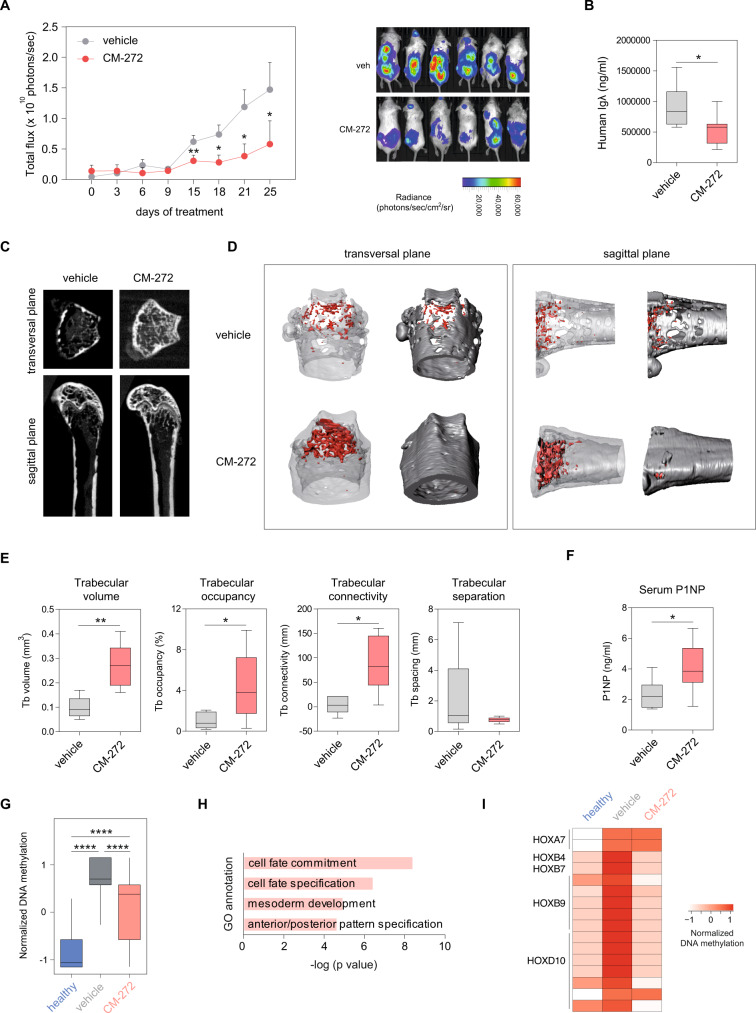
CM-272 prevents tumor-associated bone loss besides reducing multiple myeloma tumor burden. RPMI-8226-luc cells (8 × 10^6^) were intravenously injected into NSG mice. After 4 weeks, mice were randomized into 2 groups [receiving vehicle (gray) and CM-272 (red); *n* = 6/group] and treated for additional 4 weeks with dosing and regimen schedules as specified in Supplementary Methods. Tumor dissemination was checked by **A** bioluminescence measurement and **B** serum levels of human Igλ secreted by RPMI-8226-luc cells at specified time points. Line plots represent mean and SEM. Box plots represent median ±IQR, with whiskers representing the minimum and maximum. Statistical significance was determined utilizing paired two-tailed Student’s *t*-test (*p < 0.05, **p < 0.01). **C** Representative microCT cross-sections at the metaphyses of distal femurs in a vehicle and CM-272-treated mice in transversal (upper) and sagittal (down) planes. **D** Transversal (left) and sagittal (right) planes of corresponding 3D renderings from microCT images at distal femurs (trabecular bone in red, cortical bone in gray). **E** Trabecular bone morphometric parameters from microCT images were quantitated for trabecular bone volume, occupancy, connectivity, and separation. **F** Serum levels of the bone formation marker P1NP were quantified by ELISA. Graphs represent mean values ± SEM with whiskers representing minimum and maximum values. CM-272-treated (red) mice were compared to the vehicle group (gray), where *p < 0.05; **p < 0.01 versus the vehicle control group. **G** Box plots showing DNA methylation levels of pooled MSCs obtained from healthy, vehicle- and CM-272-treated animals corresponding to hypermethylated CpGs between healthy and tumor-bearing animals. **H** GO enrichment analysis of CpG sites undergoing DNA hypermethylation changes in vehicle-treated MSCs versus MSCs from healthy mice. **I** Heatmap showing normalized DNA methylation levels of individual CpGs at selected Homeobox loci among animal groups. Data pooled from mice (*n* = 6) for each group with sufficient RRBS coverage (≥5 valid sequencing reads per CpG). <0.01 by paired two-tailed Student’s *t*-test. **G** Box plots showing mean ± SEM, with whiskers representing minimum and maximum values, of DNA methylation levels of pooled MSCs obtained from healthy (blue), vehicle- (gray), and CM-272-treated (red) animals corresponding to hypermethylated CpGs between healthy and tumor-bearing animals. A paired two-tailed Student’s *t*-test was performed to calculate statistical significance (****p < 0.001). **H** GO enrichment analysis of CpG sites undergoing DNA hypermethylation changes in vehicle-treated MSCs versus MSCs from healthy mice. *p-*values were calculated utilizing a binomial test. **I** Heatmap showing normalized DNA methylation levels of individual CpGs at selected Homeobox loci among animal groups. The color scale ranges from white to red, representing low to high levels of DNA methylation. Data pooled from mice (*n* = 6) for each group with sufficient RRBS coverage (≥5 valid sequencing reads per CpG).

To test the effect of CM-272 in the context of MBD, we used an established murine model of bone marrow-disseminated myeloma. After equivalent engraftment of myeloma cells (RPMI-8226-luc) was verified by bioluminescence measurement, mice were treated for 4 weeks with CM-272 as described in Methods. Compared with the vehicle control group, CM-272 controlled tumor progression as measured by bioluminescence (Fig. 6A) or by serum levels of hIgλ secreted by RPMI-8226 cells (Fig. 6B). Representative microCT images at the metaphyses of distal femurs showed a tumor-associated bone loss in vehicle-treated mice, in contrast with trabecular structures observed in CM-272-treated animals (Fig. 6C). In the vehicle control group, 3D reconstruction images of distal femurs revealed a marked bone loss evidenced by a thin trabecular network (in red) but also by loss of cortical bone (in gray) in vehicle-treated mice (Fig. 6D). By contrast, CM-272-treated mice presented a gain in both trabecular and cortical bone (Fig. 6D). This was also reflected by bone morphometric parameters that resulted in increased trabecular bone volume, occupancy, and connectivity and reduced trabecular separation in CM-272-treated animals, as compared with vehicle control (Fig. 6E). Finally, these findings correlated with a significant increase in serum levels of the bone formation marker P1NP analyzed after CM-272 treatment compared to untreated control (Fig. 6F). In summary, these data demonstrate that CM-272 exerts in vivo anti-myeloma activity along with bone-anabolic effects in human MM-bearing mice.

To further examine the in vivo effect of CM-272 on DNA methylation of myeloma-associated MSCs, we performed reduced representation bisulfite sequencing (RRBS) analysis of MSCs isolated from vehicle- and CM-272-treated myeloma-bearing mice using healthy mice as controls. First, we observed significant alterations in the DNA methylome of MSCs from myeloma-bearing mice compared to healthy mice (Supplementary Data 8). Myeloma-bearing mice that were treated with CM-272 displayed a partial reversion of aberrant hypermethylation of MSCs caused by the presence of myeloma cells (Fig. 6G). These DNA methylation changes occurred at genomic loci enriched for genes involved in cell commitment and differentiation, such as Homeobox genes (Fig. 6H). Specifically, we were able to identify CpGs that experienced a gain in DNA methylation in vehicle-treated MSCs compared to healthy controls at the same genomic loci previously identified in human MM-MSCs, including *HOXA7*, *-B4*, *-B7*, *-B9,* and *-D10* (Fig. 6I and Supplementary Data 9). Importantly, CM-272 treatment was able to restore the DNA methylation levels at these loci to resemble that of healthy mice, which was concomitant with the reduced tumor burden as well as bone loss recovery observed in these mice (Fig. 6I).

## Discussion

The pathogenic transition from premalignant stages to active MM is complex and not well understood. One example of this complexity is that although all MM cases emerge from the pre-existing asymptomatic MGUS/SMM stage, not all MGUS progress into MM and may exist as a stable and independent disease. Nevertheless, despite being an asymptomatic stage, transformed plasma cells in MGUS present cytogenetic alterations similar to that of myeloma plasma cells, as well as significant abnormalities in bone remodeling^49,50^. This indicates that both genetic and microenvironmental alterations exist from the early stages of the disease. In our study, we show that epigenetic alterations in MSCs already occur in the early asymptomatic stages of both MGUS and SMM, and although many alterations are shared between all stages, the majority of DNA methylation changes are specific to each stage. These results are in accordance with previous studies that indicate the existence of stage-specific epigenetic alterations during MM progression in malignant plasma cells^51,52^. This phenomenon could be explained by the expansion of sub-populations of MSCs during MM disease progression, which may favor tumor development and drug resistance, similar to what was observed to occur in MM cells^53,54^.

Deregulation of methylome in MM-MSCs mediates transcriptional and phenotypical alterations. Interestingly, many genes of the Homeobox family displayed both epigenetic and transcriptional dysregulation in patient MSCs, and these changes were observed in earlier stages of the disease. In this regard, members of the HOX family have been recently described to be key drivers of OB differentiation, in which their expression is fine-tuned by demethylation of their promoters during the osteogenic process^55^. Furthermore, we observed that healthy MSCs exposed to MM cells, similarly to that observed in patient MSCs, not only displayed an altered methylome but also showed impaired MSC-to-OB differentiation, as previously described^20^. We also observed that some of these methylome changes in MSCs occur in the absence of direct cell–cell contact with MM cells, suggesting the contribution of secretory mechanisms. Hence, our results suggest that the impairment of osteogenesis in all stages of MM arises from early transcriptional deregulation of Homeobox genes, and altered DNA methylation may be the primary mediator in this process. Nevertheless, we cannot overlook the limitations of our in vitro studies, as other cell types of the BM microenvironment may also play important roles in perpetuating the methylome alterations observed in MSCs.

Although the biology of MBD is relatively well described, there is still a lack of pharmacological treatments to improve bone loss. Clinically approved bone-modifying agents for the treatment of MBD include bisphosphonates^56^, which inhibit bone resorption by suppressing OC activity, and denosumab^57^, a monoclonal antibody against the osteoclastogenic cytokine RANKL. However, these drugs only target the OC compartment, and bone disease persists due to the absence of bone formation. Thus, therapeutic agents targeting OBs are needed. In this study, we demonstrated a strategy for treating MBD by targeting aberrant DNA methylation in MSCs. Firstly, we showed that co-culture of healthy MSCs with MM cell lines yielded epigenetic and transcriptional changes similar to that observed for MSCs from myeloma patients, and treatment with CM-272 was able to at least partially reverse these changes. Additionally, this agent promoted the ability of MSCs to differentiate into OBs. These in vitro effects on bone were mirrored in a mouse model of disseminated MM. Of note, CM-272 treatment not only prevented bone loss by bone-anabolic effects but also showed anti-myeloma activity. This is in line with previous reports showing that DNMTs are targets for the treatment of MM^58–60^ and also for improving the osteogenic differentiation ability of MSCs^61^. Additionally, we cannot discard the possibility that the observed effects on tumor growth inhibition may be a consequence of the impairment of the cross-talk between MSCs and MM cells. Moreover, the dual targeting effects of CM-272 also inhibit the dimethylation of H3K9, which has been described to be crucial in the establishment of DNA methylation^42,43^. It is therefore rational to envision that the bone-anabolic effects mediated by CM-272, both in vitro and in vivo, involves the reversion of aberrant hypermethylation at Homeobox loci and other OB-related genes in the MSC population. Nevertheless, it is possible that reduced tumor burden could be partially responsible for restoring the bone-forming capacities of MM-MSCs.

In summary, our findings highlight the existence of aberrant DNA methylation patterns in the BM-derived MSC population which may impact myeloma progression and the development of MBD. Moreover, our preclinical results support the idea that therapeutic targeting of aberrant DNA methylation would result in an anti-myeloma effect and preservation of the appropriate osteogenic differentiation of MSCs to combat myeloma bone disease.

## Methods

### Participants

BM samples were obtained from the iliac crest of patients with newly diagnosed MGUS (*n* = 10), SMM (*n* = 8), and MM (*n* = 9), according to the International Myeloma Working Group criteria. BM samples from healthy controls (*n* = 8) were obtained from participants undergoing orthopedic surgery not related to oncology disease. Each sample was obtained after receiving the informed written consent of all participating subjects and following approval from the committees listed below for obtaining them and for the study protocol using them. The study was approved by the Cancer Research Center–IBMCC Review Board (CICIC 2015/02156), the Clinical Ethics Committee for drug research in the Salamanca Health Area (CEIC 73/07/2015), the Clinical Research Committee of the Bellvitge University Hospital (ref. PR076/15) and the Research Ethics Committee of the University of Navarra (ref. 2017.218). Clinical characteristics of MGUS, SMM, and MM patients are listed in Supplementary Table 2.

### Inhibitor

CM-272 (dual DNMTs and G9a inhibitor) was synthesized at the Center for Applied Medical Research (University of Navarra)^44,47,62^.

### Cell lines

The human multiple myeloma cell line MM.1S was provided by Dr. Steven Rosen (Northwestern University, Chicago, IL), whereas RPMI-8226 cells were purchased from the American Type Culture Collection. The human mesenchymal stem cell (MSC) line immortalized by expression of the telomerase reverse transcriptase gene (hMSC-TERT) was a generous gift from Dr. D Campana (Department of Pediatrics, Yong Loo Lin School of Medicine, National University of Singapore, Singapore). Both cell lines were cultured in RPMI 1640 medium supplemented with 10% heat-inactivated fetal bovine serum (FBS), 100 U/ml penicillin and 100 mg/ml streptomycin, and 1% l-glutamine. All the cell culture media and reagents were purchased from Invitrogen (Paisley, UK). All cell types were cultured at 37 °C in a humidified atmosphere in the presence of 5% CO_2_–95% air.

### Bone marrow-derived MSC isolation and culture

MSCs were isolated and characterized as described by Garayoa et al.^17^. Briefly, bone marrow aspirates were obtained from the iliac crest and subjected to centrifugation on Ficoll-Paque (GE Healthcare, Uppsala, Sweden) to obtain mononuclear cells (BMMCs). BMMCs were plated and plastic-adherent cells were expanded until passage 3 (P3) in low-glucose DMEM supplemented with 10% heat-inactivated fetal bovine serum, 100 U/ml penicillin, 100 mg/ml streptomycin, and 1% l-glutamine. Selected MSCs from both MM patients (*n* = 4) and healthy donors (*n* = 4) at P3 were tested to meet minimal criteria as defined by the International Society for Cellular Therapy for multipotent mesenchymal stromal cells^63^. Specifically, MSCs were evaluated by FACS for positive expression of CD73, CD90, CD105, CD44, and CD166 and negative staining for HLA-DR and hematopoietic markers (CD19, CD34, and CD45) (Supplementary Fig. 3A). In addition, the capability to differentiate into osteoblast, adipocyte, and chondrocyte was assessed (Supplementary Fig. 3B–D). Analyses and experiments were performed with MSCs at P3, with a maximum of 3 weeks at each passage.

### DNA and RNA isolation and quantification

Genomic DNA was isolated by the proteinase K method or using the Maxwell^®^ RSC Cell DNA Purification Kit (Promega) for samples containing low cell number. RNA was isolated using Maxwell^®^ RSC simplyRNA Cells Kit (Promega) according to the manufacturer’s instructions. DNA and RNA were quantified using Qubit® DNA Assay Kit (Invitrogen) or NanoDrop ND-1000, respectively.

### DNA methylation and gene expression profiling using arrays

DNA samples were bisulfite-converted using an EZ DNA methylation kit (Zymo Research, Orange, CA) and hybridized onto an Infinium^®^ MethylationEPIC BeadChip array (Illumina, Inc.). The array platform allows the assessment of DNA methylation status at >850,000 CpG sites at single-nucleotide resolution and covers 99% of RefSeq genes and 95% of CpG islands with an average number of six probes per island.

RNA samples were obtained from healthy donors (*n* = 8), MGUS (*n* = 10), SMM (*n* = 10), and MM patients (*n* = 24) at diagnosis and 100 ng of excellent quality RNA (RIN > 9) was hybridized onto a GeneChip Human Gene 1.0 ST (Affymetrix).

### Quality control, data normalization, and detection of differentially methylated and variable CpGs

Methylation array data were processed in the statistical language R v4.0 in RStudio 1.3 (https://rstudio.com) using methods from the Bioconductor libraries *minfi* (v1.36.0), *lumi* (v2.42.0), and *limma* (v3.46.0)^64–66^. Probes were annotated using *IlluminaHumanMethylationEPICmanifest* v0.3.0^67^. Data quality was assessed using the standard pipeline from the *minfi* package. The data were quantile-normalized and chromosomes X and Y were removed to avoid technical and biological bias. Furthermore, we discarded the DNA methylation changes associated with the long-term culture of BM-MSCs based on the previous studies^68^. *M* values (log_2_-transformed *β-*values) were utilized to obtain a *p*-value between sample groups by an eBayes-moderated paired *t*-test using the *limma* package, in which age and sex were added in the interaction matrix. For the analysis of MSCs isolated from MM patients and healthy controls, we considered a probe to be differentially methylated when the difference between the mean of *β* at disease versus control was over 10% (Δ*β* ≥ 0.1) and the statistical test was significant (**p < 0.01). In addition, we used the iEVORA algorithm^69^, provided by *matrixTests* v0.1.9 (https://CRAN.R-project.org/package=matrixTests), to designate a probe as differentially variable. This algorithm detects the homogeneity of variances using the Bartlett’s test (FDR < 0.05) and then selects those probes whose *t*-test is significant (*p < 0.05) in order to regularize the variability test which is overly sensitive to single outliers.

To evaluate the contribution of various covariates, including age and sex, we performed either a Pearson correlation or Wilcoxon signed-rank test depending on whether the covariate of interest was continuous or categorical. This is represented in Supplementary Fig. 4, in which a covariate with a *p*-value < 0.05 was considered to significantly contribute to DNA methylation.

For the direct co-culture of healthy MSCs with MM cell line, samples were normalized utilizing Noob and Quantile normalizations provided by *minfi*. The paired analysis was performed and a probe was considered differentially methylated if Δβ was more than 10% and *p*-value was <0.01.

### Gene ontology, motif, and chromatin state analysis

Functional annotation enrichment analysis was performed using GREAT tool v4.0.4 (http://great.stanford.edu/public/html)^70^ by mapping differentially methylated CpG site to the single nearest gene. CpGs annotated in the EPIC 850K array were used as background. GO categories with *p*-value of <0.01 were considered significantly enriched.

For TF binding motif analysis, HOMER motif discovery software v4.5 was used^27^, where a 500 bp-window upstream and downstream of the differentially methylated CpG sites was applied. CpGs annotated in the EPIC array were used as background.

To analyze chromatin states associated with DMPs, ChromHMM^26^ data sets from healthy donor MSCs were downloaded from the UCSC Genome Browser (https://genome.ucsc.edu/). Overlap was performed in R using the *GenomicRanges* package v1.42.0^71^, where CpGs annotated in the EPIC array were used as background.

### Gene expression array normalization and analysis

Data processing and normalization were carried out using the R statistical language. Background correction was performed using Robust Microarray Analysis (RMA) normalization provided by *oligo* package v1.54.1^72^ and probes were annotated utilizing the *hugene10sttranscriptcluster.db* R package v8.7.0^73^. Average expression was calculated for probes mapping to the same gene. For comparisons between groups, eBayes-moderated *t*-test provided by the *limma* R package^65^ was applied, where a *p*-value < 0.05 was considered statistically significant. DMPs were mapped to the nearest gene utilizing the GREAT online tool, and overlap with differentially expressed genes were performed by overlapping gene names.

### Bisulfite pyrosequencing

For total DNA extraction, cells were lysed using lysis buffer (50 mM Tris pH 8.8, 10 mM EDTA pH 8.3, 100 mM NaCl, 1% SDS) in the presence of Proteinase K (Roche). Repeated centrifugation was performed to separate nucleic acids from lipids, in which DNA was subsequently precipitated using isopropanol and washed with 75 % ethanol. 100-300 ng of isolated DNA were bisulfite (BS)-converted using EZ DNA Methylation-Gold™ Kit (Zymo Research, CA, USA) according to manufacturers’ instructions. BS-converted DNA (~10 ng) was used as a template for amplification by conventional PCR using IMMOLASE^TM^ DNA Polymerase kit (Bioline, London, UK). PCR primers were designed with the PyroMark Assay Design v2.0.2 software (Qiagen). PCR products were pyrosequenced with the PyromarkTM Q24 system (Qiagen), according to the manufacturer’s protocol.

### Quantitative real-time PCR (qRT-PCR)

Reverse transcription was performed using the Transcriptor First Strand cDNA Synthesis Kit (Roche) according to the manufacturer’s instructions. qRT-PCR was performed using LightCycler® 480 II System with LightCycler® 480 SYBR Green Mix and data were analyzed with LightCycler® 480 II Software, version 1.5, all provided by Roche. Reactions were performed in triplicate for both the target and the housekeeping gene ribosomal protein L38 (*RPL38*) used for normalization. Relative quantification of the target gene expression was calculated by the comparative threshold cycle (Ct) method.

### Co-culture system and MSC sorting

MSCs from healthy donors at passage 3 (8 × 10^3^ cells/cm^2^) or the hMSC-TERT cell line (10 × 10^3^ cells/cm^2^) were first cultured in 100 mm culture dishes until they reached ~85% confluency, and then MM.1S cells (1:3 MSC:MM.1S ratio) were added in RPMI 1640 medium supplemented with 10% FBS and antibiotics. MM cells were changed twice a week until day 14 when MSCs were recovered by trypsinization and flow cytometry-based sorting of CD13^+^ cells (BD Biosciences). For transwell experiments, MSCs from healthy donors were seeded on bottom chambers and MM.1S and RPMI-8226 cells were seeded on PET membrane inserts containing 1 μm size pores to allow an exchange of soluble molecules. Transwell experiments were performed as in direct co-cultures. For OB differentiation studies, MSCs from healthy donors were cultured in an osteogenic medium supplemented with 20% of conditioned media from the MM.1S cell line. This medium was changed twice a week until day 10 (ALP activity) or day 20 (OB mineralization). For the isolation of mouse MSCs, cells were stained with a combination of CD45-PE, Ter-119-PE, Sca-1-FITC, and PDFGRα-APC (BD Biosciences) as previously reported^74^ (Supplementary Fig. 5). Cell sorting experiments were performed by the Flow Cytometry Core Facility at Germans Trias i Pujol Research Institute utilizing FACSAria II cell sorter and analyzed using BD FACSDiva version 6.1.1 (BD Biosciences, San Jose CA).

### OB differentiation assays

OBs were generated from mesenchymal precursors by culture in osteogenic medium (containing 5 mM β-glycerophosphate and 50 mg/ml ascorbic acid) and assayed as in Garcia-Gomez et al.^75^. Briefly, primary MSCs (P2–3) were cultured in osteogenic medium for the analysis of alkaline phosphatase (ALP) activity, expression of osteogenic markers (day 10), and formation of mineralized-nodules formation (day 20). ALP activity was determined by hydrolysis of *p*-nitrophenylphosphate (Sigma-Aldrich) into p-nitrophenol and NBT/BCIP substrates (Roche), whereas mineralization was assessed by quantitative measurement of Alizarin Red (Sigma-Aldrich) staining and absorbance was measured by Multiskan Sky Microplate Spectrophotometer via SkanIt PC software (ThermoFisher).

### MTT assay

MSCs were seeded in 96 well plates and treated with increasing concentrations of CM-272. MTT was added at a final concentration of 0.5 mg/ml and incubated at 37 °C for 1 h. Cells were then washed with PBS and incubated in the dark for 10 min in the presence of dimethyl sulphoxide. Absorbance at 570 nm was measured utilizing the Multiskan Sky Microplate Spectrophotometer.

### Flow cytometry antibodies

Prior to analysis and sorting by flow cytometry, distinct amounts of MSCs were stained with fluorochrome-conjugated antibodies. Antibody specifications and concentrations used were the following: CD13-PE (BD Biosciences, 347406), CD45-PE (eBioscience, 12-0451-81), Ter-119-PE (eBioscience, 12-5921-81), Sca-1-FITC (eBioscience 11-5981-81), PDFGRα-APC (BD Biosciences, 562777), CD44-FITC (BD Biosciences, 347943), CD19-PerCP (BD Biosciences, 332780), CD90-FITC (BD Biosciences, 555595), HLA-DR-PerCP (BD Biosciences, 347402), CD14-FITC (BD Biosciences, 345784), CD166-PE (BD Biosciences, 559263), Cd45-PercCPcy5.5 (BD Biosciences, 332784), CD34-FITC (Invitrogen, 11-0349-42), CD73-PE (BD Biosciences, 550257), CD105-APC (R&D System, FAB10971A).

### Chromatin immunoprecipitation (ChIP)-quantitative PCR

MSCs (15 × 10^3^ cells per IP) were cross-linked with 1% formaldehyde for 15 min and subjected to chromatin immunoprecipitation after sonication. ChIP-qPCR assays were performed using LowCell ChIP kit^™^ protein A (Diagenode) and the antibody (5 μg) against H3K9me2 (H3K9me2 Abcam ChIP-grade, clone:mAbcam 1220, Ref:ab1220, Lot:GR45436-1). Data are represented as the ratio of the bound fraction over the input for each histone modification or factor. IgG was used as a negative control. Primer sequences were designed as close as possible from the CpG undergoing methylation changes. Primer sequences are shown in Supplementary Table 3. These experiments were performed with three biological replicates of each origin.

### In vivo model

Animal experiments were conducted according to relevant ethical regulations for the use of laboratory animals and after acquired permission from the University of Salamanca Committee for animal experimentation (ref # 0000061). BALB/c-*Rag2*^null^ *IL2rγ*^null^ (BRG) mice // or NOD-*scid IL2rγ*^null^ (NSG) mice were bred and maintained in the SPF area of the University of Salamanca Animal Facility with controlled environment conditions (20–23 °C, 12:12 light/dark cycles, 30–70% relative humidity) and fed ad libitum. CM-272 was solubilized in 0.9% saline solution. RPMI-8226-luc cells (8 ×10^6^) were injected intravenously into 8-week-old NOD-SCID-IL-2Rγ^−/−^ (NSG) mice (Charles River Laboratories) and tumor development was monitored by noninvasive bioluminescence imaging (BLI) with a Xenogen IVIS 50 system (Caliper Life Sciences). After 4 weeks, animals were randomized into two groups (*n* = 6/group) receiving vehicle (0.9% saline solution) or CM-272 (5 mg/kg, 5 times/week by intraperitoneal injection).

### Microcomputed tomography analysis

One femur of each animal was fixed in 10% formalin in order to preserve bone microarchitecture. 3D X-ray tomographic images were acquired using a Quantum-GX microCT (Perkin Elmer) with the following parameters: 80 kVp X-ray source voltage, 120 μA current, and the high-resolution scan protocol for a total acquisition time of 14 min and a gantry rotation of 360 degrees. The tomographic three-dimensional images containing the entire bone yielded a total of 512 slices, with isotropic 50 microns voxel size and a resolution of 512 × 512 pixels per slice. To perform the bone histomorphometry analysis a (10 × 10 × 10 mm) ROI containing the bone metaphysis was defined and subsequently reconstructed from the original scan at a resolution of 20 microns per voxel using the Quantum 3.0 software.

Analysis of trabecular microarchitecture in the distal femur was carried out using ImageJ v1.8.0^76^. First of all, cortical and trabecular bones were segmented from the CT volume. To this end, the following steps were followed: (i) segmentation of the entire bone volume by thresholding the original volume to obtain a 3D binary mask; (ii) segmentation of empty volumes inside the cortical volume (trabecular-free zones) using logical operators over filled vs. unfilled versions of the result of step i; (iii) segmentation of the interior volume of the cortical bone by applying 10 morphological dilations followed by 10 morphological erosions to the 3D mask obtained in step ii; (iv) segmentation of the cortical bone by performing an XOR logical operation between the masks obtained in steps i and iii; and finally, (v) segmentation of the trabecular bone by performing an AND logical operation between the masks obtained in steps i and iii. The final cortical and trabecular bone segmentations were further refined by applying a median filter to remove noise in the respective 3D masks. Cortical and trabecular bone volumes were then calculated by applying the segmentation masks on the original volume.

From the obtained trabecular masks, histomorphometry parameters were calculated using the BoneJ plugin (version 1.4.2)^77^. Finally, bone 3D reconstruction and visualization were performed using Amira 5.2 software (ThermoFisher Scientific).

### ELISA

Serum levels of human Igλ (indicating tumor burden) and N-terminal propeptide of type I procollagen (P1NP) (indicating bone formation) were measured in mice sera using the Human Lambda ELISA kit (Bethyl Laboratories, Texas, USA) and Rat/Mouse PINP EIA kit (Immunodiagnostic Systems, East Boldon, UK), respectively, following manufacturers’ instructions. Absorbance was measured using the Multiskan Sky Microplate Spectrophotometer.

### Reduced representation bisulfite sequencing (RRBS)

Sorted MSCs from three groups (healthy, vehicle-treated, and CM-272-treated mice) were pooled in order to obtain a significant number of cells for performing the RRBS-seq. Isolated DNA from each pool was subjected to the RRBS pipeline as previously described^78^. In brief, purified DNA was digested with MspI and subjected to bisulfite conversion. Following PCR amplification, RRBS libraries were generated from sequenced DNA following previously published procedures (http://code.google.com/p/bsmap/downloads/). Downstream normalization and analyses were performed following a previously published pipeline (http://rrbs-techdev.computational-epigenetics.org/). CpG annotation and GO enrichment analysis was performed utilizing the GREAT online tool.

### Primers

All primers used are listed in Supplementary Table 3.

### Statistical analysis

Data are expressed as mean ± SEM and the *n* value for each in vitro assay is specified in the corresponding figure legend. Statistical analyses were carried out with Prism version 6.0 (GraphPad) and were performed using a two-tailed Mann–Whitney *U* test or Student’s *t*-test.

### Reporting summary

Further information on research design is available in the Nature Research Reporting Summary linked to this article.

## Supplementary information

Supplementary Information

Description of Additional Supplementary Files

Supplementary Data 1–9

Reporting Summary

## Data Availability

The DNA methylation and expression data supporting the findings of this study have been deposited in the NCBI’s Gene Expression Omnibus database under the accession code GSE137419. This SuperSeries (GSE137419) is composed of the following SubSeries: GSE137360 (methylation), GSE137369 (expression), and GSE137416 (methylation II). All the other data supporting the findings of this study are available within the article and its supplementary information files and from the corresponding author upon reasonable request. Source data are provided with this paper.

## Source data

Source Data
