# Supplementary material for: Targeting aberrant DNA methylation in mesenchymal stromal cells as a treatment for myeloma bone disease
Source: Nat Commun. 2021 Jan 18;12:421. doi: 10.1038/s41467-020-20715-x (PMC7813865; doi:10.1038/s41467-020-20715-x)
Supplement: Supplementary file 1 — Supplementary Information [file 41467_2020_20715_MOESM1_ESM.pdf]

Supplementary Figure 1

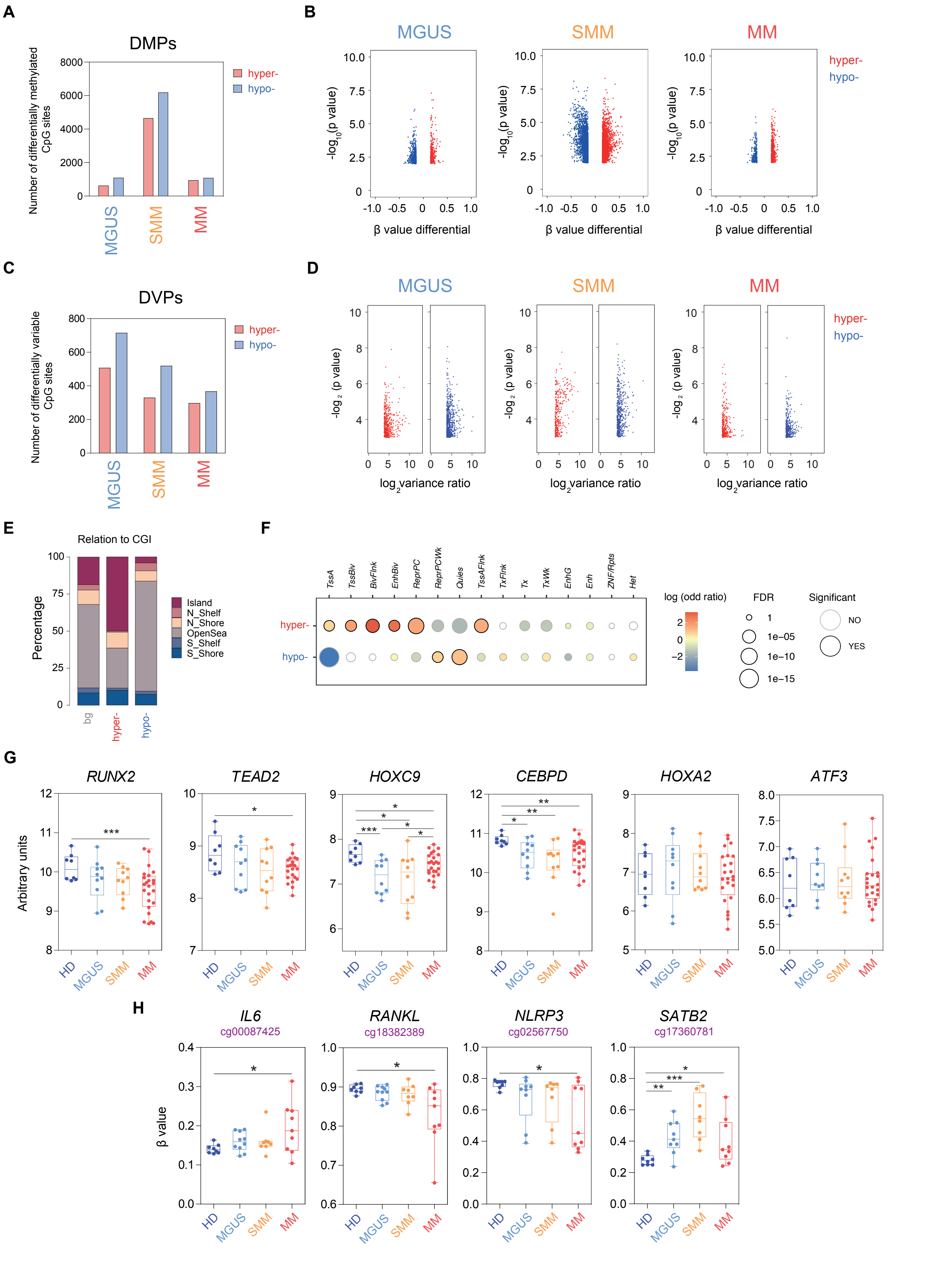

Supplementary Figure 1. Analysis of differentially methylated (DMPs) or variable (DVPs) CpG positions in BM-derived MSCs from MGUS, SMM and MM patients.
A) Bar plots showing the number of hyper- (red) and hypo- (blue) methylated CpG positions (DMPs) in each of the MM stages compared to healthy controls. B) Volcano plots showing difference of mean methylation versus the significance of the difference in the comparisons between MGUS, SMM and MM samples and healthy individuals ($\Delta\beta \geq 0.1$ and $^{**}p < 0.01$) for hyper- (red) and hypomethylated (blue) DMPs. C) Bar plots showing the number of differentially hyper- (red) and hypo- (blue) variable CpG positions (DVPs) in each of the MM stages compared to their healthy counterparts. D) Volcano plots showing the p value vs the variance ratio for each of the MM stages-associated DVPs ($q < 0.05$ and $^{*}p < 0.05$). E) Percentage of accumulative hyper- and hypo-variable CpGs that fall within a CpG island (CGI), north and south shelf or shore, and open sea. CpGs annotated in the EPIC array was used as background (bg). F) Enrichment analysis of DVPs located in different genomic regions, annotated by 15 chromHMM states. Color scale refers to log odd ratio and circle size refers to p value significance. G) Box plots showing β-values obtained from the EPIC array in MSCs from healthy donors and MGUS , SMM and MM patients of relevant genes involved in the pathogenesis of MM and associated bone disease. H) Box-plots showing expression data obtained from the Human Gene 1.0 ST array (Affymetrix) of transcription factors previously identified in HOMER analysis in MSCs from healthy donors and MGUS/SMM/MM patients. ANOVA t-tests were performed for G and H where $^{*}p < 0.05$, $^{**}p < 0.01$ and $^{***}p < 0.001$. Analysis was performed with healthy donors ($n = 8$) and MGUS ($n = 10$), SMM ($n = 8$) and MM ($n = 9$) patients.

Supplementary Figure 2

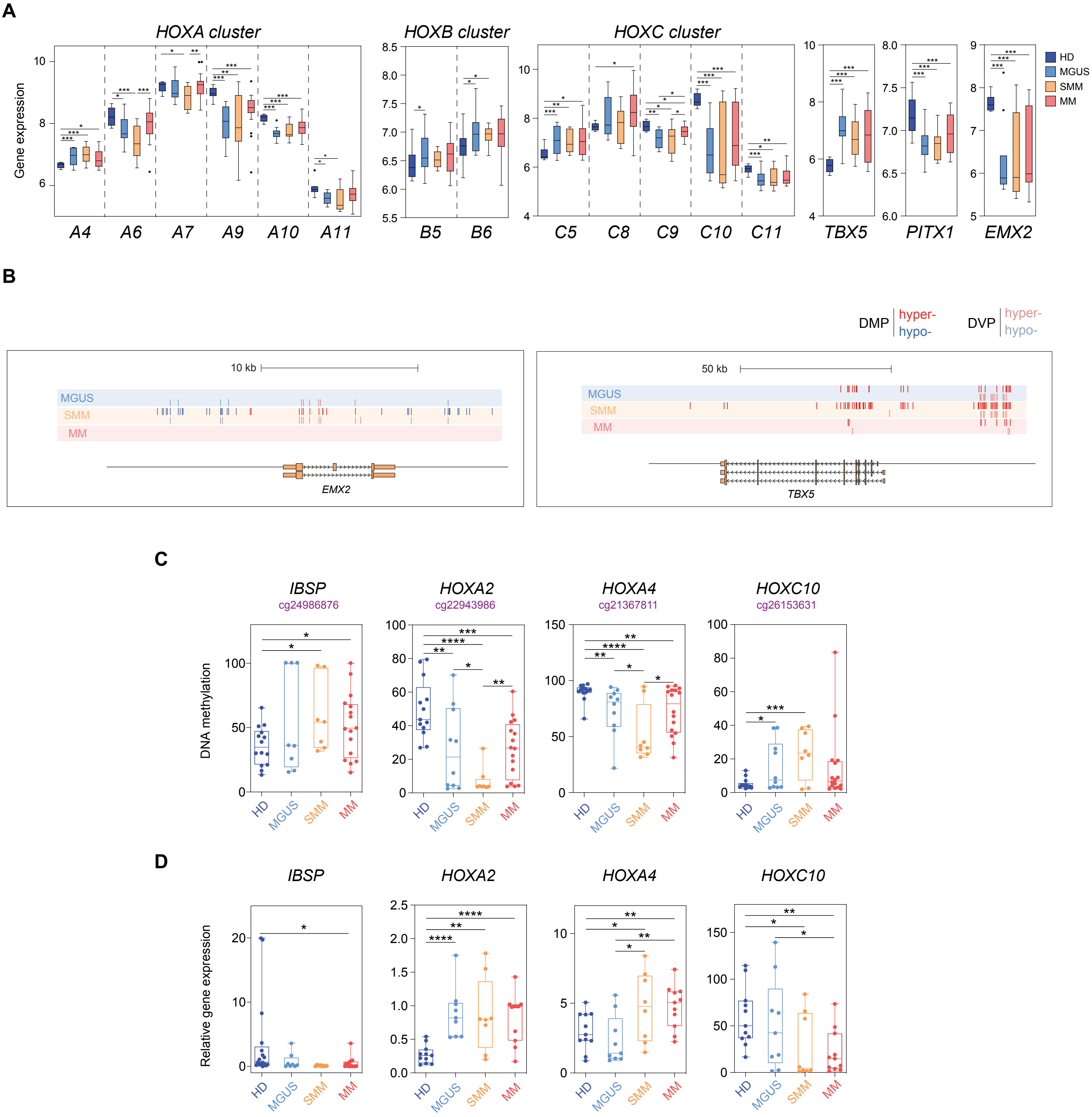

Supplementary Figure 2. Representation of differentially expressed and methylated Homeobox genes in BM-derived MSCs from healthy controls and MGUS, SMM and MM patients.

A) Box plots showing expression data obtained from the Human Gene 1.0 ST array (Affymetrix) of differentially expressed Homeobox genes in MSCs from healthy donors (dark blue; n = 8) and MGUS (light blue; n = 10), SMM (orange; n = 8) and MM (red; n = 9) patients.

ANOVA t-test was performed to determine statistical significance (*p < 0.05, **p < 0.01 and ***p < 0.001).

B) Scheme depicting differentially methylated and variable CpG sites located in the Homeobox genes (EMX2 and TBX5). Dark blue lines indicate hypo-methylated DMPs, light blue lines indicate hypo-methylated DVPs, dark red lines indicate hyper-methylated DMPs and light red lines indicate hyper-methylated DVPs associated to MGUS, SMM and MM conditions.

C) DNA methylation and D) gene expression levels analyzed by qRT-PCR of IBSP, HOXA2, -A4 and -C10 genes in MSCs from healthy controls (n = 17), MGUS (n = 8), SMM (n = 8) and MM (n = 16) patients, as normalized against RPL38.

Statistical significance was calculated using two-sided unpaired student t-tests (*p value < 0.05, **p value < 0.01 and ***p value < 0.001).

Supplementary Figure 3

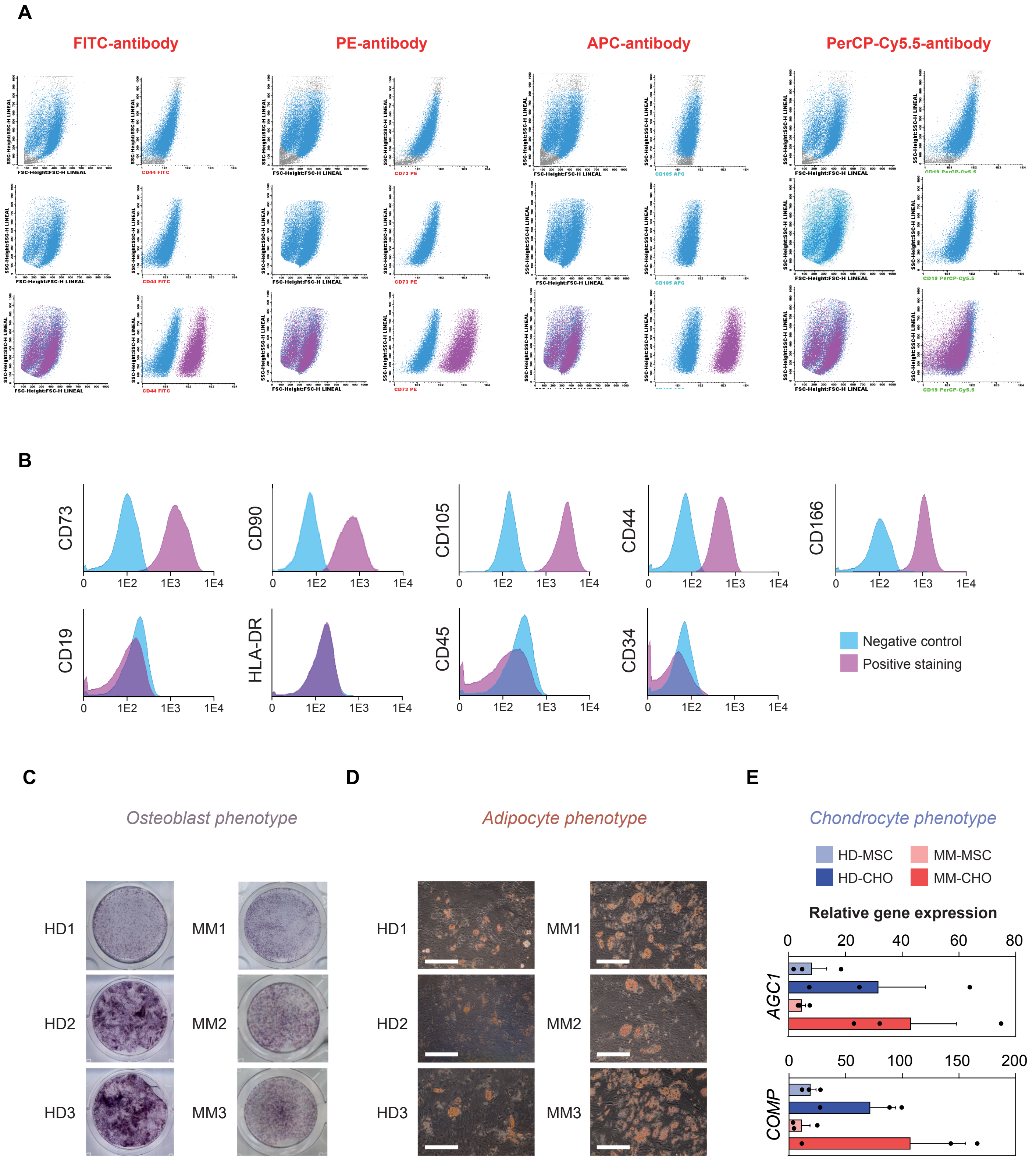

Supplementary Figure 3. Human MSC characterization.
A) Representative examples of gating strategies of FITC, PE, APC and PerCP-Cy5.5. Gray points represent cell debris, blue represents whole cells and purple represents positive staining. B) Representative example of immunophenotypic analysis of positive (CD73, CD90, CD105, CD44, CD166) and negative (CD19, HLA-DR, CD45, CD34) markers used to characterize the bone marrow MSC population. Negative control refers to unstained MSCs. C) Images of alkaline phosphatase activity in differentiated OBs from mesenchymal precursors of 3 healthy donors (HD1-3) and 3 MM patients (MM1-3) performed by NBT-BCIP staining. D) Images of lipid droplet accumulation in differentiated adipocytes from MSCs of 3 healthy controls (HD1-3) and 3 MM patients (MM1-3) performed by Oil Red O staining. Bar represents 250μM. E) Gene expression levels of chondrocyte markers AGC1 and COMP in differentiated chondrocytes (darker shade) from mesenchymal precursors (lighter shade) of healthy donors (blue) and MM patients (red). Bar graphs represent the mean and SEM of 3 independent experiments.

Supplementary Figure 4

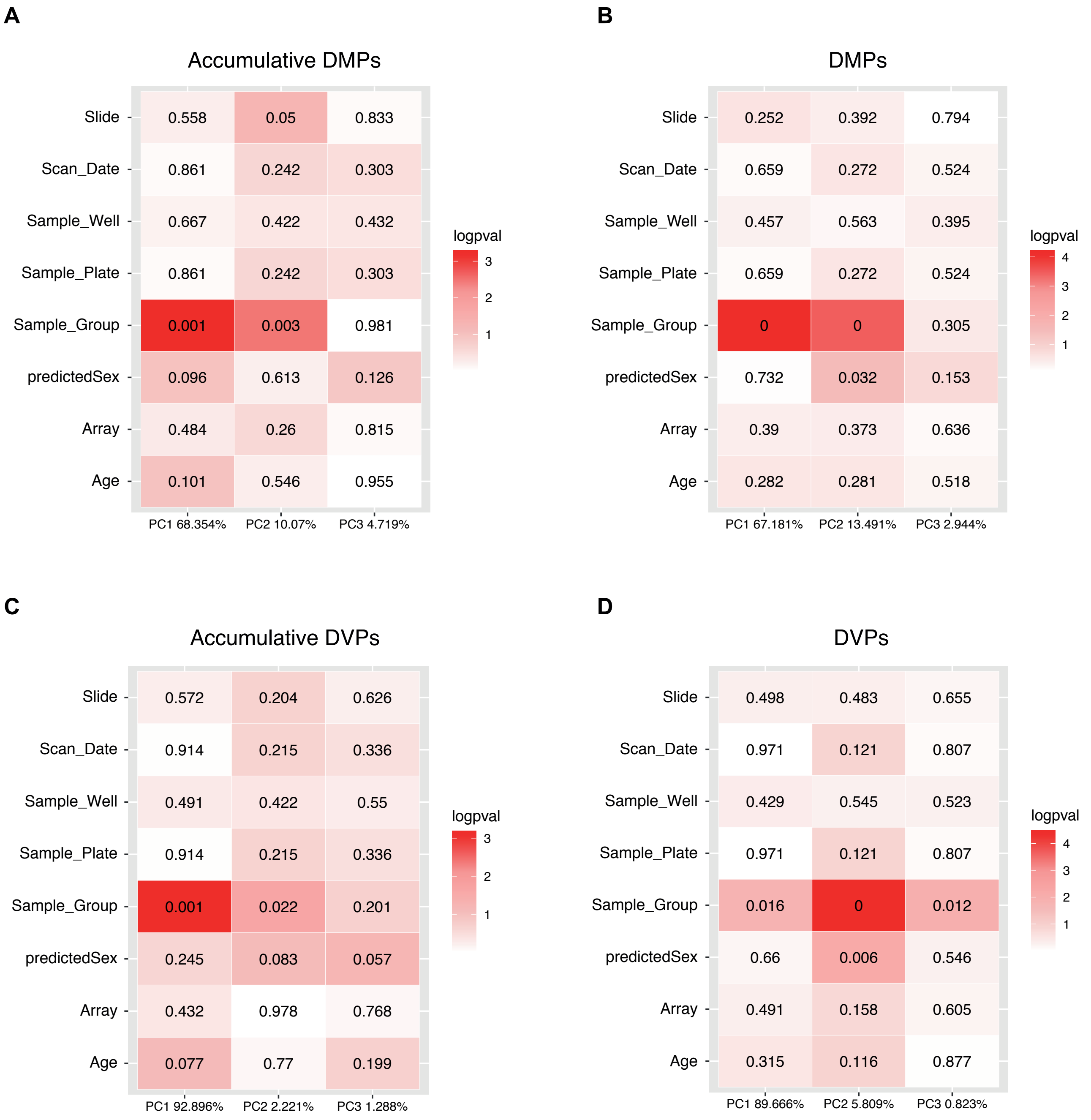

Supplementary Figure 4. Pearson correlation or Wilcoxon signed-rank test was performed to evaluate the contribution of continuous or categorical covariates respectively to DNA methylation of identified accumulative.
A) DMPs, B) DMPs, C) accumulative DVPs and D) DVPs. Contribution of each Principal Component (PC) to total variance is shown as a percentage. Color scale from white to red represents less to more significant p-values. To calculate statistical significance, Pearson correlation or Wilcoxon signed-rank test was performed depending on whether the covariate of interest was continuous or categorical.

Supplementary Figure 5

A

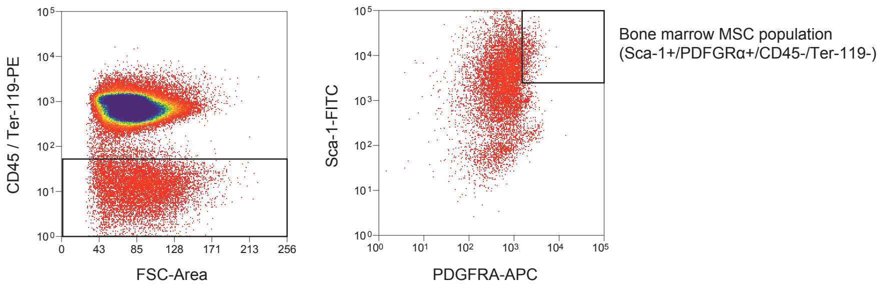

Supplementary Figure 5. Sequential gating strategy used for the isolation of mouse MSCs.
A) Positive cells for CD45 and Ter-119 (hematopoietic markers) were excluded from further analysis. Mouse MSCs were selected on the basis of expression of Sca-1 and PDGFR-α.

Supplementary Table 1. Selected genes that are both dysregulated at the DNA methylation and expression levels in MSCs from distinct MM stages

Gene	Relevance in osteoblast differentiation and function
RUNX2	master transcription factor of osteoblastogenesis; its expression and activation is impaired in MM
EMX2	promotes OB differentiation
IRX5	loss disrupts cranial mineralization
FGFR2	positive regulator of OB proliferation and osteoblastogenesis
HOXA10	promotes osteogenic differentiation through activation of Runx2 and direct activation of OB phenotypic genes (*ALP*, *BGLAP*, *SPP1*) in MSCs
SEMA3A	SEMA3A stimulates OB differentiation through canonical Wnt signaling
NRP2	codes for a transmembrane receptor that binds to SEMA3A to regulate bone formation
COL12A1	is involved in matrix bridge formation during osteogenesis
TCF3	positive regulator of osteogenic differentiation *in vitro* and ectopic bone formation *in vivo*
TWIST1	critical modulator of mesenchymal cell fate during skeletal development
PITX1	downregulated in OBs from adolescent idiopathic scoliosis patients compared to controls
MSX2	regulates osteogenic *vs* adipogenic differentiation from mesenchymal precursors
EN1	required for the expression of OB differentiation markers at early and late stages of calvarial osteogenesis
HOXA2	inhibits OB marker expression (*RUNX2, SP7, ALP*) via BMP modulation in palatal mesenchyme
HOXA4	HOXA4 is involved in adipocyte differentiation from mesenchymal precursors
HOXC8	negative regulator of osteogenesis
MAB21L1	OBs from mice lacking Mab21l1 displayed higher expression of osteogenic markers and mineralization
MAB21L2	codes for a repressor of BMP-induced transcription; upregulated in MSCs from osteoporotic individuals
SFRP2	MM cells with advanced bone lesions secrete SFRP2 that suppresses BMP2-induced OB differentiation
OSR1	overexpression in calvaria cells leads to decreased levels of OB markers (BGLAP, SP7)
NFATC2	its activation in OBs inhibits bone formation and causes cancellous bone osteopenia

In blue, downregulated genes; in red, upregulated genes

Supplementary Table 2. Clinical characteristics of DNA methylation cohort

	Mean or %	SD or N	P-value
Age (years)			
Controls (n = 8)	43	10.2	
MGUS (n = 10)	71.4	8.1	< 0.0001#*
SMM (n = 8)	57.4	6	0.0051#*
MM (n = 9)	71.1	9.5	0.0001#*
Female			
Controls (n = 8)	75 %	6	
MGUS (n = 10)	40 %	4	0.14†
SMM (n = 8)	62.5 %	5	0.59†
MM (n = 9)	33.3 %	3	0.09†
Bone lesion score (1-3)			
MGUS (n = 10)	0	0	
SMM (n = 8)	0	0	
MM (n = 9)	1.9	0.74	

#P-values were calculated by Mann-Whitney U test

†P-values were calculated by Chi-squared test

*Statistical significance p-value < 0.05

Supplementary Table 3. Primer list

Gene	Assay	Sense	Sequence
HOXA6	ChIP	Forward	CTTAGGAGCCCAGTCTCACG
HOXA6	ChIP	Reverse	TGAACCCAGGGAATTGAAAG
HOXA9	ChIP	Forward	CCTGTGTGGCTTCTGAAACA
HOXA9	ChIP	Reverse	CAAATCGCATTGTCGCTCTA
HOXA10	ChIP	Forward	CTCCTGGCCCATCAATACAG
HOXA10	ChIP	Reverse	CACTCCCAGTTTGGTTTCGT
HOXC9	ChIP	Forward	CTCTTCTTCACCCACCCTGA
HOXC9	ChIP	Reverse	TGCTTTGCATCAGACTGTCC
PITX1	ChIP	Forward	CTATCCCAATCCCGGAAAAT
PITX1	ChIP	Reverse	GCTTGGACCTTCTCCCCTAC
RUNX2	ChIP	Forward	AGGCCTTACCACAAGCCTTT
RUNX2	ChIP	Reverse	GTGGGACTGCCTACCACTGT
HOXA6	qPCR	Forward	AAAGCACTCCATGACGAAGG
HOXA6	qPCR	Reverse	ATGGCTCCCATACACAGCAC
HOXA9	qPCR	Forward	GCGCCTTCTCTGAAAACAAT
HOXA9	qPCR	Reverse	CAGTTGGCTGCTGGGTTATT
HOXA10	qPCR	Forward	CTCGCCGGAGAAGGATTC
HOXA10	qPCR	Reverse	TTCTTCCGACCACTCTTTGC
HOXC9	qPCR	Forward	CAGCAAGCACAAAGAGGAGA
HOXC9	qPCR	Reverse	GTAGGGGCAGCGCTTCTT
PITX1	qPCR	Forward	CCAGCGAGTCGTCTGACA
PITX1	qPCR	Reverse	CTCCCGCACCACTGTCCT
RUNX2	qPCR	Forward	CCCTGAACTCTGCACCAAGT
RUNX2	qPCR	Reverse	GGCTCAGGTAGGAGGGGTAA
HOXC10	qPCR	Forward	GACACCTCGGATAACGAAGC
HOXC10	qPCR	Reverse	CCTCTTCTTCCTTCCGCTCT
HOXC8	qPCR	Forward	GTCTCCCAGCCTCATGTTTC
HOXC8	qPCR	Reverse	CAAGGTCTGATACCGGCTGT
HOXA2	qPCR	Forward	TTGCCTCAGCCACAAAGAAT
HOXA2	qPCR	Reverse	TTGGTGTAAGCAGTTCTCAGG
HOXA4	qPCR	Forward	CCCTGGATGAAGAAGATCCA
HOXA4	qPCR	Reverse	GGTGTAGGCGGTTCGAGAG
IBSP	qPCR	Forward	CCGAAGAAAATGGAGATGACA
IBSP	qPCR	Reverse	CCTCTCCATAGCCCAGTGTT
BGLAP	qPCR	Forward	CTCACACTCCTCGCCCTATT
BGLAP	qPCR	Reverse	CGCCTGGGTCTCTTCACTAC
SPP1	qPCR	Forward	GCCGAGGTGATAGTGTGGTT
SPP1	qPCR	Reverse	TGAGGTGATGTCCTCGTCTG
HOXB7	qPCR	Forward	GCCCTTTGAGCAGAACCTCT
HOXB7	qPCR	Reverse	TCTGGTAGCGGGTGTAGGTC
HOXB9	qPCR	Forward	TAATCAAAGACCCGGCTACG
HOXB9	qPCR	Reverse	GTGTAGGGACAGCGCTTTTT
HOXD10	qPCR	Forward	CTGAGGTCTCCGTGTCCAGT
HOXD10	qPCR	Reverse	TTCTGCCACTCTTTGCAGTG
ACVR2A	qPCR	Forward	GCGTTTGCCGTCTTTCTTAT
ACVR2A	qPCR	Reverse	CAGCCAACAACCTTGTTTCA
EBF2	qPCR	Forward	GGAGTGGTGGACGCTAATGT
EBF2	qPCR	Reverse	GCCAGGACGAAGTGAAAGAA
HOXA2	Pyroseq	Forward	GGGTTTTAGGGTGTTAAAATTTTGAAGA
HOXA2	Pyroseq	Reverse	[Btn]ATAACTACCCTCTACCTCCC
HOXA2	Pyroseq	Seq	GGTGTTTAAATGATTTGTGA
HOXA4	Pyroseq	Forward	GGGTTTTGGGTTTTTAGTTAATTTTAGT
HOXA4	Pyroseq	Reverse	[Btn]TTCTCCAACTCCAAAACCTACT
HOXA4	Pyroseq	Seq	AGTTATAAAGGAGGGGAGTTTAA
HOXC8	Pyroseq	Forward	GTTTTTTTAGAGAGTGGGTAGGAGTTA
HOXC8	Pyroseq	Reverse	[Btn]ACACCAATTCTACTAATAACCTAAATTAAA

HOXC8	Pyroseq	Seq	GTGGGTAGGAGTTATG
HOXC10	Pyroseq	Forward	ATGGGGAAAAAAAAAGATGTTAGT
HOXC10	Pyroseq	Reverse	[Btn]ACTCCCAAACTACATATACATACC
HOXC10	Pyroseq	Seq	TTTTTTTTTTGAAAATGATATGTT
HOXA9	Pyroseq	Forward	GGATTGTTTTAGTTGAGGGTATGG
HOXA9	Pyroseq	Reverse	[Btn]CCAAATCCAAACATCCCCTCATCA
HOXA9	Pyroseq	Seq	GTTAATAGGAGAGTAGGAA
HOXA6	Pyroseq	Forward	[Btn]AGGGTATTTAGGGAGGTTTTAAG
HOXA6	Pyroseq	Reverse	AACACAATATACCCAACCACTTCCTTTTAA
HOXA6	Pyroseq	Seq	ACCACTTCCTTTTAAACTT
HOXA10	Pyroseq	Forward	GGGTTTATTTAGAAGGAGTATTTAGTGA
HOXA10	Pyroseq	Reverse	[Btn]CCCAACTACTAAATTTCTCCCTCTC
HOXA10	Pyroseq	Seq	ATTTAGTGAATTGTAGTTTAATTTT
HOXC9	Pyroseq	Forward	[Btn]GGGGGAGGAATTTAAGTTGTTTGATA
HOXC9	Pyroseq	Reverse	CTCCTTTACCCCCAACTCCATCTCTA
HOXC9	Pyroseq	Seq	CTTTCATTAAATAAAATTATAAACT
RUNX2	Pyroseq	Forward	GTATGTAATATTGTATTGTGGGTAGTAGTT
RUNX2	Pyroseq	Reverse	[Btn]ATTTCTTAAATCCTTTTATACTAATTTACT
RUNX2	Pyroseq	Seq	ATGTTTTTTATATTTTTTGTAAGT
PITX1	Pyroseq	Forward	TGAGTTTTAGGGAGAAGAGGTTGGAGAA
PITX1	Pyroseq	Reverse	[Btn]ACACACCTCAAACCTCAAATAACTT
PITX1	Pyroseq	Seq	GGTAGAGAAGGGAGTTGATTTTAG
HOXB7	Pyroseq	Forward	[Btn]GGTTTTGAAATTAAATTTTGATTTGTTT
HOXB7	Pyroseq	Reverse	ATCTCCCAAAATACTAAAATTACAAATT
HOXB7	Pyroseq	Seq	CTAAATACTATATACAAAAACTAAC
HOXB9	Pyroseq	Forward	[Btn]GTAAGGAATGGGGTTGTTTTT
HOXB9	Pyroseq	Reverse	CCAAATCTAACTAATTTTCAAAAAATACT
HOXB9	Pyroseq	Seq	ACTCCCAAACTAATAAATCAAATCC
HOXD10	Pyroseq	Forward	AGGAGGAGAGAAGAGATAAGTGAG
HOXD10	Pyroseq	Reverse	[Btn]ACCTACTCTAAAAAATCAAACCATTATC
HOXD10	Pyroseq	Seq	ATATTTTTTTTTTTATTTAGGGGTA
ACVR2A	Pyroseq	Forward	AGAGAATTTTATGATATTGGTAGGTATA
ACVR2A	Pyroseq	Reverse	[Btn]TCCTAAAACAACCAAATTTTTATCTCTCAA
ACVR2A	Pyroseq	Seq	GTAGGTATATAATTTTTTTTAGTAT
EBF2	Pyroseq	Forward	TTTAGAATTTTGATAGAGTGGGGTTTTT
EBF2	Pyroseq	Reverse	[Btn]ACATTCCTACCCAAAACCTATACA
EBF2	Pyroseq	Seq	GATGGTTGTGTGGTT
HOXA3	Pyroseq	Forward	GTAAGAATTTGTTAGGGGAAGGG
HOXA3	Pyroseq	Reverse	[Btn]ATCTTAAAATCCAAACCCCACTTCTACC
HOXA3	Pyroseq	Seq	TTTTGGGTTAAGGGG
IBSP	Pyroseq	Forward	GAGTTAATGAAGTATATTTTAGGAGGAAA
IBSP	Pyroseq	Reverse	[Btn]AATTCTAAAACCAAAAAATTTACATTAT
IBSP	Pyroseq	Seq	AATTATATTTAATGATAGTAAGTTT
BGLAP	Pyroseq	Forward	ATGAGTAGGGTAGGGTTTGAGT
BGLAP	Pyroseq	Reverse	[Btn]ACCCCCAACCTCAAATTAAACAC
BGLAP	Pyroseq	Seq	TTGAATTTATTTTAGGTTTTTGA
