# Supplementary material for: Targeting aberrant DNA methylation in mesenchymal stromal cells as a treatment for myeloma bone disease
Source: Nat Commun. 2021 Jan 18;12:421. doi: 10.1038/s41467-020-20715-x (PMC7813865; doi:10.1038/s41467-020-20715-x)
Supplement: Supplementary file 2 — Description of Additional Supplementary Files [file 41467_2020_20715_MOESM2_ESM.pdf]

Description of Additional Supplementary Files

Supplementary Data 1-9: .ZIP folder containing:

Supplementary Data 1: List of differentially methylated CpG positions (DMPs) in BM-derived MSCs from MGUS, SMM and MM patients compared to healthy controls.

Supplementary Data 2: List of differentially variable CpG positions (DVPs) in BM-derived MSCs from MGUS, SMM and MM patients compared to healthy controls.

Supplementary Data 3: List of differentially methylated CpG positions associated to MM progression (accumulative DMPs).

Supplementary Data 4: List of differentially variable CpG positions associated to MM progression (accumulative DVPs).

Supplementary Data 5: List of differentially expressed genes in BM-derived MSCs from MGUS, SMM and MM patients.

Supplementary Data 6: List of differentially methylated and expressed genes in BM-derived MSCs from MGUS, SMM and MM patients.

Supplementary Data 7: List of differentially methylated CpGs in healthy MSCs co-cultured with the MM.1S cell line and overlapping with MGUS/SMM/MMassociated DMPs and/or DVPs.

Supplementary Data 8: List of all analyzed CpGs in MSCs from healthy, vehicle-treated and CM-272-treated mice.

Supplementary Data 9: List of hypermethylated DMPs in MSCs from MMbearing compared to healthy mice overlapping with hypermethylated DMPs found in MSCs of MGUS/SMM/MM patients compared to HDs.
