# Supplementary material for: Targeting aberrant DNA methylation in mesenchymal stromal cells as a treatment for myeloma bone disease
Source: Nat Commun. 2021 Jan 18;12:421. doi: 10.1038/s41467-020-20715-x (PMC7813865; doi:10.1038/s41467-020-20715-x)
Supplement: Supplementary file 4 — Reporting Summary [file 41467_2020_20715_MOESM4_ESM.pdf]

nature research

Corresponding author(s): Ballestar, Esteban

Last updated by author(s): 02/12/2020

Reporting Summary

Nature Research wishes to improve the reproducibility of the work that we publish. This form provides structure for consistency and transparency in reporting. For further information on Nature Research policies, see Authors & Referees and the Editorial Policy Checklist .

Statistics

For all statistical analyses, confirm that the following items are present in the figure legend, table legend, main text, or Methods section.

n/a	Confirmed	
☐	☒	The exact sample size (*n*) for each experimental group/condition, given as a discrete number and unit of measurement
☐	☒	A statement on whether measurements were taken from distinct samples or whether the same sample was measured repeatedly
☐	☒	The statistical test(s) used AND whether they are one- or two-sided<br>*Only common tests should be described solely by name; describe more complex techniques in the Methods section.*
☐	☒	A description of all covariates tested
☐	☒	A description of any assumptions or corrections, such as tests of normality and adjustment for multiple comparisons
☐	☒	A full description of the statistical parameters including central tendency (e.g. means) or other basic estimates (e.g. regression coefficient) AND variation (e.g. standard deviation) or associated estimates of uncertainty (e.g. confidence intervals)
☐	☒	For null hypothesis testing, the test statistic (e.g. *F*, *t*, *r*) with confidence intervals, effect sizes, degrees of freedom and *P* value noted<br>*Give P values as exact values whenever suitable.*
☒	☐	For Bayesian analysis, information on the choice of priors and Markov chain Monte Carlo settings
☒	☐	For hierarchical and complex designs, identification of the appropriate level for tests and full reporting of outcomes
☒	☐	Estimates of effect sizes (e.g. Cohen's *d*, Pearson's *r*), indicating how they were calculated

Our web collection on statistics for biologists contains articles on many of the points above.

Software and code

Policy information about availability of computer code

Data collection	Methylation and gene expression array data were processed in the statistical language R. DNA methylation and gene expression array data were obtained from Infinium® MethylationEPIC BeadChip array (Illumina, Inc.) and GeneChip Human Gene 1.0 ST (Affymetrix) respectively. Raw data was processed using R statistical software v4.0 ran in RStudio 1.3 (https://rstudio.com).
Data analysis	DNA methylation data was normalized using minfi v1.36.0 and probes were mapped using IlluminaHumanMethylationEPICmanifest v0.3.0. Gene expression data was normalized using oligo v1.54.1 and probes were annotated using hugene10sttranscriptcluster.db v8.7.0. Group comparisons were performed using limma v2.42.0. DVPs were calculated using the iEVORA algorithm provided by matrixTests v0.1.9. Gene ontology analysis of DNA methylation was performed using the online GREAT tool v4.0.4 and TF motif enrichment was performed using HOMER motif discovery software v4.5. Genomic overlaps were performed using GenomicRanges package v1.42.0. Primers for pyrosequencing experiments were designed using PyroMark Assay Design v2.0.2 software and DNA methylation was analyzed using PyromarkTM Q24 system. qRT-PCR experiments were analyzed using LightCycler® 480 II Software, version 1.5 and Statistical analyses were carried out with Prism version 6.0 (GraphPad). Cell sorting experiments were performed using the program BD FACSDiva version 6.1.1. For mineralization experiments, Alizarin Red quantification was performed using SkanIt PC software (Thermo Fisher). Image analyses of mice models were performed using Xenogen IVIS 50 system (Caliper Life Sciences), ImageJ v1.8.0, BoneJ plugin v.4.2 and Amira 5.2 software (Thermo Fisher).

For manuscripts utilizing custom algorithms or software that are central to the research but not yet described in published literature, software must be made available to editors/reviewers. We strongly encourage code deposition in a community repository (e.g. GitHub). See the Nature Research guidelines for submitting code & software for further information.

Data

Policy information about availability of data

All manuscripts must include a data availability statement. This statement should provide the following information, where applicable:

- Accession codes, unique identifiers, or web links for publicly available datasets
- A list of figures that have associated raw data
- A description of any restrictions on data availability

DNA methylation and expression data supporting the findings of this study have been deposited in NCBI's Gene Expression Omnibus with the accession number GSE137419.

Field-specific reporting

Please select the one below that is the best fit for your research. If you are not sure, read the appropriate sections before making your selection.

[x] Life sciences [ ] Behavioural & social sciences [ ] Ecological, evolutionary & environmental sciences

For a reference copy of the document with all sections, see nature.com/documents/nr-reporting-summary-flat.pdf

Life sciences study design

All studies must disclose on these points even when the disclosure is negative.

Sample size	No sample size calculation was performed. For in vitro experiments, MSCs from at least 3 independent donors were used, which allowed sufficient data to perform paired student t-tests to determine statistical significance. For mice models, six mice were decided for each experimental group. We believe that this number provided sufficient statistical power, with relatively low standard deviation, for group comparisons. For donor samples, a minimum of 8 donors were recruited in each group. Although this is a relatively small number, it allowed us to generate a linear weighted model for ANOVA analysis. Furthermore, we believe that this number of patients is representative of a larger population.
Data exclusions	No data was excluded for the analysis.
Replication	Methylation data was validated in an independent cohort of primary MSCs from healthy donors and patients at different MM stages. All attempts at replication were successful.
Randomization	Samples allocation was not random, as each sample comes from a known specific diagnosis. The effect of each known covariates were first calculated using either Pearson correlation or Wilcoxon signed-rank test depending on whether the covariate of interest was continuous or categorical. In the cases where samples were not matched, specific covariates were imputed in the interaction matrix when generating linear weighted models for group comparisons. Sample hybridization was randomized to minimize batch effects.
Blinding	Blinding was performed during data collection of microCT samples. Blinding was not possible during data collection, as our experimental design involved the recruitment of age- and sex-matched patients from various dignostic stages of MM.

Reporting for specific materials, systems and methods

We require information from authors about some types of materials, experimental systems and methods used in many studies. Here, indicate whether each material, system or method listed is relevant to your study. If you are not sure if a list item applies to your research, read the appropriate section before selecting a response.

Materials & experimental systems

n/a	Involved in the study	
[ ]	[x]	Antibodies
[ ]	[x]	Eukaryotic cell lines
[x]	[ ]	Palaeontology
[ ]	[x]	Animals and other organisms
[ ]	[x]	Human research participants
[x]	[ ]	Clinical data

Methods

n/a	Involved in the study	
[x]	[ ]	ChIP-seq
[ ]	[x]	Flow cytometry
[x]	[ ]	MRI-based neuroimaging

Antibodies

Antibodies used	CD13-PE BD (clone:L138, Ref:347406, Lot:8123555, Dil:2μL/Million cells), CD45-PE eBioscience (clone:30-F11, Ref:12-0451-81, Lot:2051673, Dil:1μL/Million cells), Ter-119-PE eBioscience (clone:TER-119, Ref:12-5921-81, Lot:2023946, Dil:2μL/Million cells), Sca-1-FITC eBioscience (clone:D7, Ref:11-5981-81, Lot:1989493, Dil:2μL/Million cells), PDFGR -APC BD (clone:APA5, Ref:562777, Dil:2μL/Million cells), CD44-FITC BD (clone:L178, Ref:347943 Lot:7103691, Dil:2μL/Million cells), CD19-PerCP BD

	(clone:SJ25C1, Ref:332780, Lot:7184879, Dil:2µL/Million cells), CD90-FITC BD (clone:5E10, Ref:555595, Lot:7170536, Dil:1µL/Million cells), HLA-DR-PerCP BD (clone:L243, Ref:347402, Lot: 6266788, Dil:1µL/Million cells), CD14-FITC BD (clone:MyP9, Ref: 345784, Lot:27517, Dil:2.5µL/Million cells), CD166-PE BD (clone:3A6, Ref:559263, Lot: 4255806, Dil:1µL/Million cells), Cd45-PercCPcy5.5 BD (clone:2D1, Ref:332784, Lot:6195827, Dil:1µL/Million cells), CD34-FITC Invitrogen (clone: 4H11, Ref:11-0349-42, Lot:1984086, Dil:1µL/Million cells), CD73-PE BD (clone:AD2, Ref:550257, Lot:3319691, Dil:2µL/Million cells), CD105-APC R&D System (clone:166707, Ref:FAB10971A, Lot:AAHU216011, Dil:2µL/Million cells), H3K9me2 Abcam (ChIP-grade, clone:mAbcam 1220, Ref:ab1220, Lot:GR45436-1, Dil:4µl/Million cells).
Validation	CD13-PE was validated as indicated on the official webpage of BD Biosciences (https://www.bdbiosciences.com/eu/reagents/clinical/reagents/single-antibodies/cd13-pe-l138-also-known-as-leu-m7/p/347406). Furthermore, the expression of CD13 in MSCs has been previously confirmed by FACS by Muñiz et al. (PMID: 26347461).<br>The antibody combination for mouse bone marrow MSC identification was validated in previous studies (PMID: 23154782).

Eukaryotic cell lines

Policy information about cell lines

Cell line source(s)	The human multiple myeloma cell line MM.1S was provided by Dr. Steven Rosen (Northwestern University, Chicago, IL). MM.1S cell line was established from peripheral blood of a multiple myeloma patient who had become resistant to steroid-based therapy. hMSC-TERT cell line was a generous gift from Dr. D Campana (Department of Pediatrics, Yong Loo Lin School of Medicine, National University of Singapore, Singapore) and was established by ectopic expression of TERT from the bone marrow of a healthy donor. RPMI-8226 cells were purchased from the American Type Culture Collection.
Authentication	MM.1S and RPMI-8226 cell lines were authenticated as criteria of ATCC and hTERT-MSC cell line was authenticated as experiments performed (PMID:12614220).
Mycoplasma contamination	Cell lines tested were negative for mycoplasma contamination.
Commonly misidentified lines (See ICLAC register)	No commonly misidentified cell lines were used in this study.

Animals and other organisms

Policy information about studies involving animals; ARRIVE guidelines recommended for reporting animal research

Laboratory animals	14 mice of 8-week-old NOD-SCID-IL-2Rγ-/- (NSG) of female sex were used in the study. BALB/c-Rag2null IL2rγnull (BRG) mice // or NOD-scid IL2rγnull (NSG) mice were bred and maintained in the SPF area of the University of Salamanca Animal Facility with controlled environment conditions (20-23ºC, 12:12 light/dark cycles, 30-70% relative humidity) and fed ad libitum. All animal work was conducted according to relevant national and international guidelines for animal research and approved by the Bioethics Committee of the University of Salamanca (reg # 0000061).
Wild animals	Study did not involve wild animals.
Field-collected samples	Study did not involved samples colleted from the field.
Ethics oversight	Animal experiments were conducted according to institutional guidelines for the use of laboratory animals and after acquired permission from the local Ethical Committee for animal experimentation.

Note that full information on the approval of the study protocol must also be provided in the manuscript.

Human research participants

Policy information about studies involving human research participants

Population characteristics	Bone marrow samples were obtained from newly diagnosed patients of MGUS, SMM and MM. Bone marrow samples from healthy controls were obtained from participants undergoing orthopedic surgery. Clinical characteristics of MGUS, SMM and MM patients are listed in Supplementary Table 2.
Recruitment	Patients from each MM stage were recruited according to the International Myeloma Working Group criteria. Healthy donors were sex-matched, but not age-matched. To correct the effect of age on DNA methylation analyses, age was included as a covariate in the interaction matrix to generate linear models for group comparisons.
Ethics oversight	Each sample was obtained after receiving informed written consent of all participating subjects and following approval from the University Hospital of Salamanca de Salamanca Review Board.

Note that full information on the approval of the study protocol must also be provided in the manuscript.

Flow Cytometry

Plots

Confirm that:

- [x] The axis labels state the marker and fluorochrome used (e.g. CD4-FITC).
- [x] The axis scales are clearly visible. Include numbers along axes only for bottom left plot of group (a 'group' is an analysis of identical markers).
- [x] All plots are contour plots with outliers or pseudocolor plots.
- [x] A numerical value for number of cells or percentage (with statistics) is provided.

Methodology

Sample preparation	Human samples were collected by trypsinization and stained according to Method section, while mice samples were collected from femurs and tibiae of each group according to specified protocol (PMID: 23154782).
Instrument	MoFlo Astrios (Beckman Coulter).
Software	Samples were analyzed using Kaluza software.
Cell population abundance	Purity was determined by the percentage of CD13+ cells (~5%) after the co-culture system in human samples.<br>In mice samples, purity was determined by the percentage of PDGFRalpha+Sca1+CD45-Ter119- cells (~0.5%).
Gating strategy	For human MSCs, cells were gated by size (FSC/SSC) and viability (negative for DAPI staining). Positive cells for CD13 marker were sorted.<br>For mouse MSCs, positive cells for CD45 and Ter-119 (hemopoietic markers) were excluded. Sorted cells were selected on the basis of expression of Sca-1 and PDGFR-α.

- [x] Tick this box to confirm that a figure exemplifying the gating strategy is provided in the Supplementary Information.
